# Metal Ion-Enhanced ZIC-cHILIC StageTip for N-Glycoproteomic and Phosphoproteomic Profiling in EGFR-Mutated Lung Cancer Cells

**DOI:** 10.1016/j.mcpro.2025.100957

**Published:** 2025-03-26

**Authors:** Yi-Ju Chen, Yan-Lin Chen, Kun-Hao Chang, Hsiang-Chun Cheng, Chiao-Chun Chang, Yu-Ju Chen

**Affiliations:** 1 Institute of Chemistry, Academia Sinica, Taipei, Taiwan; 2 Department of Chemistry, National Taiwan University, Taipei, Taiwan; 3 Molecular Science and Technology Program, Taiwan International Graduate Program, Academia Sinica, Taiwan; 4 Department of Chemistry, National Tsing-Hua University, Hsinchu, Taiwan

**Keywords:** ZIC-cHILIC, glycoproteomics, phosphoproteomics, TKI resistant, NSCLC cell

## Abstract

Surface glycosylation and intracellular phosphorylation regulates the cell–cell communication and signaling cascades. Due to complex glycosylation and dynamic phosphorylation, exploring their interplay remains technically challenging. In this study, we reported a tandem ZIC-cHILIC StageTip strategy for streamlined and simultaneous (sialo)glycoproteomic and phosphoproteomic profiling. We first demonstrated that Fe ions expand the utility of ZIC-cHILIC strategy to phosphoproteomic analysis with greatly enhanced >4-fold coverage and high specificity for monophosphopeptides (95%). The Fe-ZIC-cHILIC tandem tips, leveraging stepwise fractionation, enable large-scale coverage of 10,536 glycopeptides, including highly confident 4285 sialoglycopeptpides, and 11,329 phosphopeptides in a single cell type. To study the mechanism underlying the tyrosine kinase inhibitor (TKI) resistance in non–small cell lung cancer (NSCLC), application of the strategy to four NSCLC cells harboring different epidermal growth factor receptor (EGFR) mutations reveals significantly differential 1559 glycopeptides and 1949 phosphopeptides either in EGFR mutation or TKI-resistant cells. Without protein immunoprecipitation, the approach identified FDA-approved drug targets, such as EGFR, ERBB2, MET, and integrin family members. Most prominent alterations were observed in EGFR (auto-phosphorylation Y1197 and 10 biantennary and triantennary fucosyl-sialo glycans at N603), downstream PI3K-Akt pathway (ERBB2-T1240, MET-S990/T992, AKT-S124/S126), and integrin family (sialo-fucosyl glycans), suggesting site-specific alteration between N-glycosylation and phosphorylation interplay in the TKI-resistant L858R-T790M mutant NSCLC cells. The glycoproteomic and phosphoproteomic landscape may help to unravel the complex modification alterations underlying the resistant mechanism, offering insights for improving therapeutic strategies and patient outcomes.

## Highlights

• Fe-ZIC-cHILIC for simultaneous glycoproteomics and phosphoproteomics
• Fe-ZIC-cHILIC tandem tip offers large-scale glycopeptides and phosphopeptides enrichment
• Revealing cross-talk in N-glycosylationand phosphorylation-cascade at proteome scale
• Elevated glycosylation/phosphorylation on EGFR/PI3K/MAPK pathway in TKI-resistant cells

## Graphical Abstract

**Figure:**
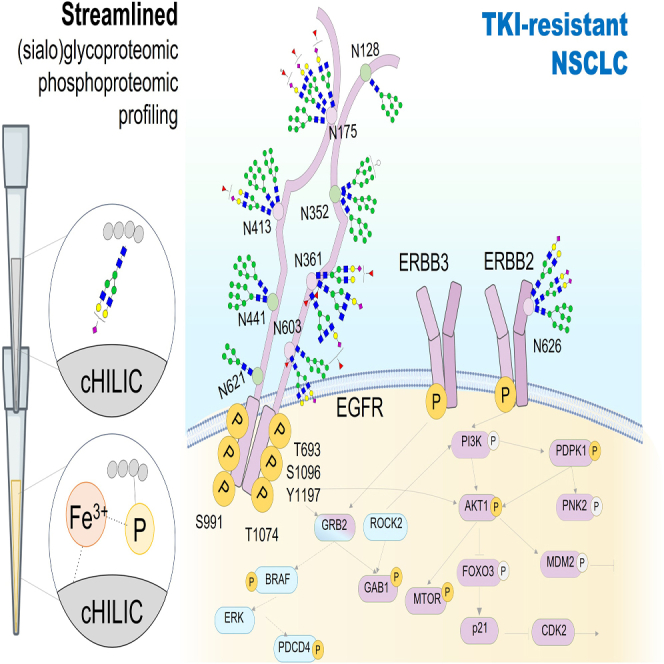

## In Brief

We reported a tandem ZIC-cHILIC StageTip strategy for streamlined and simultaneous N-glycoproteomic and phosphoproteomic profiling. This strategy provides good enrichment specificity (∼80%) and coverage of identification of 10,536 glycopeptides and 11,329 phosphopeptides from 200 μg non–small cell lung cancer cell lysate. This approach revealed FDA-approved druggable glycoproteins and their phosphorylation sites, revealing N-glycosylation and phosphorylation interplay that may correlate with EGFR mutations and resistance to tyrosine kinase inhibitor.

Post-translational modifications (PTMs) increase the diversity of protein functions and regulate a broad range of cellular biological processes (1). Extracellular glycosylation, a complex and multistep process involving around 200 enzymes (2), and intracellular phosphorylation, a fast and dynamic protein modification, controlled by kinases and phosphatases (3), are two critical PTMs that regulate diverse biological processes, often working together to influence cellular function and communication. For example, membrane proteins are extensively modified with extracellular glycosylation for ligand binding, cell–cell interactions, and immune recognition and intracellular phosphorylation to regulate downstream signaling transduction pathways. Clinically, dysregulation of membrane proteins and their PTMs have been linked to a variety of human diseases—such as cancer, neurodegenerative diseases, and other disorders—making them vital targets for biomarker discovery and therapeutic development (4, 5, 6). Deregulation of receptor tyrosine kinases (RTKs) may lead to uncontrolled cell growth and division and eventually cause cancer, presenting the most popular cancer drugs. RTKs are activated after the ligand binding and then induce the receptor dimerization (3); altered glycosylation affects the structural arrangements of RTKs, leading to unexpected cascade activation and phenotypes (4, 5). The interplay between extracellular glycosylation and intracellular phosphorylation is increasingly recognized as important in coordinating cellular responses to external stimuli. Simultaneous profiling both glycoproteome and phosphoproteome may provide a more comprehensive view of their synergistic or antagonistic interactions and how cells integrate external cues with internal regulatory mechanisms that influence disease mechanisms and treatment outcomes. The upregulated sialylated and noncore-fucosylated glycans were described to hinder the epidermal growth factor receptor (EGFR) dimerization and receptor activation, while core fucosylation was reported to promote the process (2). Despite rising interests in simultaneous characterization of glycoproteome and phosphoproteome, the low abundance of modified peptides and LC-MS/MS detectability challenged the profiling depth and sensitivity. Enrichment tools that take into account the different physicochemical properties between glycopeptides (GPs) and phosphopeptides (PPs) to achieve high recovery, selectivity, and robustness is the most critical prerequisite prior to LC-MS/MS analysis.

Due to efficient large-scale profiling, enrichment strategies were developed to extract GPs and PPs from complex peptide mixture prior to LC-MS/MS analysis. For phosphoproteomic profiling, various tools for PP enrichment have gained maturity by commonly applied immobilized metal affinity chromatography (IMAC) (7, 8, 9) or by metal-oxide affinity chromatography (9, 10). Large-scale phosphoproteomics coverage of >10,000 PP can be routinely achieved for bulk sample (11, 12) and for microscale sample (13). The conventional methods for LC-MS–based glycoproteomics included hydrophilic interaction liquid chromatography (HILIC) (14, 15), boronic acid–based materials (16), and lectin affinity approach (17, 18). Liu *et al*. have applied HILIC strategy and pGlyco2.0 software (https://github.com/pFindStudio/pGlyco2) processing to report 10,009 site-specific N-glycans on 1988 glycosylation sites from 955 glycoproteins in 5 mouse tissues (14). Zborníková *et al.* introduced the electrostatic interaction between positively charged choline groups and negatively charged analytes in zwitterionic hydrophilic interaction chromatography with the exposed choline group (ZIC-cHILIC) as powerful separation materials for complex nucleotide samples (19). We then reported the first implementation of StageTip-based ZIC-cHILIC strategy that enabled large-scale profiling of 7367 GPs, with enhanced coverage of 2742 sialo-GPs (SGPs) from 200 μg PC9 membrane fraction (Byonic score ≥100) (20). These single enrichment methods show high selectivity and profiling coverage of either the phosphoproteome or glycoproteome. However, these popular methods cannot offer information for both or other protein modifications. Considering the important cross-talking between different modifications (21, 22, 23) in the cellular signaling pathway, such as the extracellular glycosylation and intracellular phosphorylation that are spatially located on the same membrane proteome, simultaneous analysis for glycoproteomics and phosphoproteomics can help to discover PTM-mediated mechanisms.

Pioneering works reported electrostatic repulsion-hydrophilic interaction chromatography (ERLIC) and novel materials for the feasibility of simultaneous enrichment for hundreds of intact GPs and PPs (24, 25). Zheng *et al*. reported gallium-based magnetic metal-organic framework for one-pot enrichment of both N-GPs and PPs, which achieved a total of 1006 GPs and 1332 PPs in 20 μg peptides from mouse liver (26). Zhou *et al.* demonstrated dual-metal-centered zirconium–organic framework fructose-1,6-diphosphate to analyze exosome glycoproteome and phosphoproteome from HeLa cell digest, which identified 1011 N-GPs and 1996 PPs (27). Wang *et al*. reported ATP-coated dual-functional Ti-IMAC material combined with extensive fractionation strategy (6 fractions), achieving 2928 intact GPs and 3051 PPs identified from 500 μg mouse lung tissue (28). Recent work reported novel materials of tandem enrichment strategies to improve the profiling depth. Recent work by Chen *et al*. demonstrated combining Fe-IMAC and HILIC StageTips with single elution, respectively, for plant samples, which achieved identification of 1954 N-GPs and 11,255 PPs from Arabidopsis (29). Compared to the sensitivity and large-scale coverage achieved by individual enrichment strategies for the glycoproteome and phosphoproteome, the performance of their simultaneous profiling still requires significant improvement for microscale samples and higher enrichment specificity, especially to enhance the enrichment specificity of GPs.

To enable a more comprehensive and sensitive analysis of the glycoproteome and phosphoproteome, we introduce a streamlined metal ion–decorated ZIC-cHILIC strategy, featuring a tandem-tip format with single material and simple pH control, to allow simultaneous enrichment and stepwise separation of GPs and PPs. By incorporating Fe^3+^ ions onto the ZIC-cHILIC beads, for the first time, we demonstrated exceptional enrichment performance with high recovery and specificity for both GPs and PPs. This Fe-ZIC-cHILIC strategy offers straightforward material preparation using single material, and a tandem-tip which streamlines the enrichment process by reducing sample transfer steps, thereby minimizing sample loss. Tyrosine kinase inhibitor (TKI) resistance has been a significant challenge in cancer therapy since the resistance reduced the efficiency of drugs and further limited the therapy options. The PTMs play important roles in the complicated mechanism of TKI resistance. Glycosylation and phosphorylation regulate key signaling pathways, including the EGFR pathway in lung cancer. Abnormal glycosylation can affect receptor stability and ligand binding, while aberrant phosphorylation may lead to the activation of alternative pathways that bypass the effects of EGFR inhibitors. To uncover the PTM-related molecular mechanisms driving resistance and identify new therapeutic targets for more effective treatment strategies, we further applied this approach to explore the interplay between glycoproteomic and phosphoproteomic profiles in four different non–small cell lung cancer (NSCLC) cells with sensitivity or resistance in response to the EGFR inhibitor. The glycoproteomic and phosphoproteomic landscape may help to unravel the complex molecular alterations underlying the resistant mechanism, offering insights that could improve therapeutic strategies and patient outcomes.

## Experimental Procedures

### Experimental Design and Statistical Rationale

For each experiment, three technical replicates were conducted to evaluate the reproducibility and performance between different enrichment conditions. Each fraction from the sample was subjected to LC-MS/MS analysis, respectively. The average and SD of identification numbers from each sample were plotted in the bar chart. The average and SD of specificity from each sample were plotted in the scatter plot. For quantitative comparison between four NSCLC cells, the differential expression of N-GPs and PPs were calculated by label-free quantitation and the statistical significance was evaluated by Student *t* test (*p* < 0.05).

### Materials (Chemicals, Cell Lines, and Instruments)

Acetonitrile (ACN) and ZIC-cHILIC columns (3 μm, 100 Å, 250 × 4.6 mm) were acquired from MerckMillipore. Tris(2-carboxyethyl) phosphine hydrochloride, iodoacetamide, triethylammonium bicarbonate (TEABC), TFA, and formic acid (FA) were procured from Sigma-Aldrich. LC-MS/MS grade trypsin was obtained from Promega. The bicinchoninic acid protein assay kit was sourced from Pierce. Deionized water was obtained using a Milli-Q Ultrapure Water Purification System (Millipore). The styrenedivinylbenzene ne copolymer (SDB-XC) membrane (diam. 47 mm) was purchased from Millipore (Millipore Corporation).

### Cell Culture

Four cell lines of human non–small cell lung adenocarcinoma (NSCLC) cells with different EGFR subtypes—including PC9 (EGFR-Del19), CL68 (Del19 and T790M), H3255 (L858R), and H1975 (L858R and T790M)—were used in this study. All of the cell lines were cultured in RPMI-1640 medium supplemented with fetal bovine serum (10% v/v), 1 mM sodium pyruvate, sodium bicarbonate (2% w/v), 100 units/ml penicillin, and 100 μg/ml streptomycin at 37 °C in a humidified atmosphere of 5% CO_2_ in an incubator.

### Cell Lysis and Protein Extraction

PC9 cells were washed three times with cold PBS buffer and lysed by adding 2 ml of lysis buffer, containing final concentration of 12 mM SDS, 12 mM sodium deoxycholine, 0.1 M Tris–HCl (pH 9.0), 1/100 volume of a protease inhibitor, and 1/100 volume of phosphatase inhibitors (PP2 and PP3). After cell harvesting, the lysate was collected into two 2 ml eppendorfs and heated at 95 °C for 5 min, followed by sonication at 4 °C for 15 min to degrade DNA and RNA. Subsequently, the cell lysate was transferred into 15 ml centrifugation tubes. MeOH (4× volume of cell lysate), CHCl_3_ (1× volume of cell lysate), and deionized water (3× volume of cell lysate) were added to the cell lysate sequentially. After vortexing, the cell lysate was centrifuged at 3220*g* at room temperature for 15 min, resulting in the protein layer remaining. MeOH (3× volume of cell lysate) was added again to the tube, and the tubes were centrifuged at 3220*g* at room temperature for 5 min. After discarding the supernatant, the pellets were dissolved in 8 M urea solution, followed by protein quantification utilizing the bicinchoninic acid assay.

### Protein Denaturation and Digestion

For complete protein alkylation and reduction, the sample concentration should be below 2 μg/μl. Proteins were reduced by a final concentration of 5 mM tris(2-carboxyethyl) phosphine hydrochloride in 50 mM TEABC at 29 °C for 30 min and subsequently alkylated by a final concentration of 20 mM iodoacetamide at 29 °C for 30 min. After diluted 8 M urea to 1 M using 50 mM TEABC solution, the digested enzyme trypsin was added into the samples at a ratio of 1:50 (w/w), and the digestion process was allowed to proceed for 16 to 18 h. After digestion, the samples were acidified using a final concentration of 0.5% TFA solution to quench enzyme-mediated reactions, aiming for a final pH value of approximately 2.0.

### Intact N-GP Enrichment with ZIC-cHILIC StageTip

To perform GPs enrichment, homemade ZIC-cHILIC StageTips were first prepared. A frit membrane was cut by using a No. 19 syringe and placed into D200 tips by plunging it in. Holes were then made in the caps of 1.5 ml eppendorfs, and the packed tips were placed on top of the caps. ZIC-cHILIC beads (40 mg) were weighed and suspended in 100 μl of deionized water for each StageTip. Next, 100 μl of the bead solution was added into each D200 tip and centrifuged at 3000 rpm to carry out packing beads for 6 min at room temperature. The D200 tips were then rotated 180 degrees and centrifuged at 5000 rpm for 2 min at room temperature. Tryptic digests were dried and redissolved in an 80% ACN/0.5% TFA solution, with the peptide concentration adjusted to 1 μg/μl. The ZIC-cHILIC StageTips were activated by using 100 μl of 80% ACN/0.5% TFA solution twice, following by centrifuging at 5000 rpm for 4 min at room temperature and equilibration with centrifugation at 5000 rpm for 2 min at room temperature. The tips were then transferred to new 1.5 ml eppendorfs, and peptides were loaded into the ZIC-cHILIC StageTips, followed by centrifugation at 3000 rpm for 20 min. The samples were reloaded and centrifuged again at 4000 rpm for 16 min. For washing, 100 μl of 80% ACN/0.5% TFA was added twice to the ZIC-cHILIC StageTips, followed by centrifugation at 4000 rpm for 8 min and 5000 rpm for 6 min, respectively. The tips were then transferred to new 1.5 ml eppendorfs. GPs were eluted using stepwise elution, which is carried out by three fractions: 70%, 65%, and a combination of 60%-55%-50% of ACN/H_2_O solutions. Each step of elution is centrifugation at 4000 rpm for 10 min. The eluted GPs were dried by vacuum SpeedVac and ready for LC-MS/MS analysis.

### PP Enrichment With Fe-ZIC-cHILIC StageTip

In this procedure, the protocol is similar to the ZIC-cHILIC strategy mentioned above. The only difference is that Fe^3+^ ions were chelated on ZIC-cHILIC beads instead of traditional ZIC-cHILIC beads for PPs enrichment. To make Fe^3+^ decoration onto ZIC-cHILIC beads, 40 mg commercial ZIC-cHILIC beads were soaked in 100 μl of 50 mM FeCl_3_ solution for 16 h in the dark. Then Fe-coupled ZIC-cHILIC beads were suspended properly and loaded into D200 tips. Tryptic digests (100–200 μg) were dried and redissolved into 200 μl of 80% ACN/0.1% TFA. The activation, condition, sample loading steps were all the same as mentioned above. One hundred microliters of 80% ACN/0.1% TFA was added twice into the Fe-ZIC-cHILIC StageTip for washing and rewashing steps, combined with centrifugation at 4000 rpm for 8 min and 5000 rpm for 6 min, respectively. Then, the tips were transferred into new 1.5 ml eppendorfs. The captured peptides (*i.e.*, PPs) were performed by stepwise elution with three fractions: 70%, 65%, and a combination of 60%-55%-50% of ACN/H_2_O solutions. The elution conditions are the same as mentioned above.

The eluted PPs were further desalted by SDB-XC StageTip to avoid the remaining metal ions. SDB-XC StageTip was prepared by using a No.16G syringe to cut the two-layer SDB-XC membrane and using a plunger to push the membrane into the stage tip. The SDB-XC was preconditioned by adding 200 μl of buffer B (80% ACN/0.1% TFA) and centrifuged at 1000*g* for 1 min, and further added 200 μl of buffer A (5% ACN/0.1% TFA) and centrifuged at 1000*g* for 1 min for equilibrium. Peptides were loaded into StageTips and centrifuged at 500*g* for 5 min. After washing by adding 200 μl of 5% ACN/0.1% TFA and centrifuged at 1000g for 1 min, finally, peptides were eluted by 100 μl of 5% ACN/0.1% TFA. The desalted PPs were dried by vacuum SpeedVac and ready for LC-MS/MS analysis.

### Tandem cHILIC StageTip for Simultaneous Enrichment of Intact N-GPs and PPs

The innovative tandem cHILIC StageTip was coupled by ZIC-cHILIC StageTip and Fe-ZIC-cHILIC StageTip. The incubation condition, such as 80% ACN with 0.1%, 0.5%, and 1% of TFA or FA, was optimized before two-tip conjugation. The order of tandem tips was also investigated. The optimal protocol is similar to the Fe-ZIC-cHILIC strategy that was under 80% ACN/0.1% TFA. The protocol including StageTip activation, equilibrium, washing, and elution is the same with the above mentioned. At the sample loading, two tips were coupled together and 200 μg peptides were loaded and centrifuged at 1600*g* for 20 min at room temperature. Subsequently, the sample was reloaded into the tandem StageTip and centrifuged at 1700*g* for 17 min at room temperature. After washing, the GPs and PPs from the captured tips were individually eluted by three fractions with 70%, 65%, and a combination of 60%-55%-50% of ACN/H_2_O solutions. The peptides eluted from ZIC-cHILIC StageTip were dried by vacuum SpeedVac and ready for LC-MS/MS analysis. The peptides eluted from Fe-ZIC-cHILIC StageTip were desalted by SDB-XC StageTip, dried by vacuum SpeedVac, and ready for LC-MS/MS analysis.

### PP Enrichment With Fe-IMAC Stage Tip

The Fe-IMAC StageTip was prepared by cutting the frit and pushing it into the D200 StageTip. Nickel-nitrilotriacetic acid beads were dissolved using 200 μl of 6% acetic acid (AA) at pH 3.0. During the bead loading step, 25 μl of nickel-nitrilotriacetic acid beads were added and centrifuged at 2250*g* for 2 min. Subsequently, Ni^+^ ions were captured using 50 mM EDTA in 1 M sodium chloride. To chelate Fe^3+^ ions with NTA, 100 mM FeCl_3_ in 6% AA at pH 3.0 was added and also equilibrated by using 6% AA. The predesalted peptides were loaded and flow-through was reloaded to enhance the enrichment efficiency. Each step was followed by centrifugation at 500*g* for 4 min. To remove most of the nonspecific binding, 80% ACN/6% AA pH 3.0 were used. Finally, PPs were eluted by 200 mM ammonium dihydrogen phosphate by centrifugation at 500*g* for 2 min. For desalting of PPs, the experimental procedures were following the same procedure as mentioned above.

### LC-MS/MS Analysis

Dried peptide samples were redissolved in 6 μl 0.5% aqueous FA solution. After vortexing, the samples were centrifuged under 13,500 rpm for 5 min Five microliters of the sample was transferred to the vial and waited for LC-MS/MS analysis. For each injection, 4 μl of the sample was injected into Orbitrap Fusion Lumos Tribrid Mass Spectrometer (Thermo Fisher Scientific) by Ultimate 3000 RSLC nano system (Thermo Fisher Scientific) coupled with a C18 column (Thermo Fisher Scientific PepMap C18, 25 cm × 75 μm ID column with 1.9 μm C18 beads, Thermo Fisher Scientific). Buffer A (0.1% aqueous FA solution) and buffer B (0.1% FA in 100% ACN solvent) were used for the composition of the mobile phase.

Each sample was performed by 120-min data-dependent acquisition-mode LC-MS/MS analysis with the flow rate of 300 nl/min under column temperature of 42 °C. For PPs analysis, the gradient of buffer B was set as below: 2 to 5% in the first 2 min, 5 to 30% in 86 min, 30 to 50% buffer B in 4 min, 50 to 90% buffer B in 5 min, keeping 90% buffer B in 7 min, 90 to 2% buffer B in 2 min, and keeping 2% buffer B until the end of analysis. The mass scanning range was set from *m/z* 400 to 1250 in orbitrap with 120,000 resolution and automatic gain control (AGC) target of 4 × 10^5^ charges for a maximum injection time of 50 ms. The higher-energy collisional dissociation mode was set with 30% collision energy. The fragment ions scanning *via* quadrupole was performed at 30,000 resolution with an AGC target value of 5e4 charges in a dynamic maximum injection time.

For intact GP analysis, the gradient of buffer B was set as below: 2 to 5% in 2 min, 5 to 30% in 91 min, 30 to 50% in 4 min, 50 to 90% in 2 min, 90 to 2% in 1 min, and keeping 2% until the end of analysis. The MS1 range was set from *m/z* 400 to 2000 in orbitrap with 120,000 resolution and AGC target of 4e5 charges for maximum injection time in 50 ms. Stepped higher-energy collisional dissociation mode was set with 27%, 35%, and 43% collision energy. The MS/MS scanning *via* quadrupole was performed with 30,000 resolution in orbitrap with an AGC target of 5e4 charges in dynamic maximum injection time.

### Data Processing

After mass spectrometry analysis, all raw files were analyzed in Proteome Discoverer 2.5 software (https://www.thermofisher.com/us/en/home/industrial/mass-spectrometry/liquid-chromatography-mass-spectrometry-lc-ms/lc-ms-software/multi-omics-data-analysis/proteome-discoverer-software.html; Thermo Fisher Scientific) integrating with SEQUEST search engine. The fasta file Swissprot_homosapiens_2021128 (with 20,291 protein sequences) was imported from the UniProt website as the protein database. For PP analysis, the carbamidomethyl of cysteine was set as the static modification, while protein N-terminal acetylation, deamination at asparagine and glutamine, and phosphorylation on serine (S), threonine (T), and tyrosine (Y) were set as the dynamic modifications. The PP identification was performed using the following parameters: maximum of two missed cleavages, maximum of four modifications, peptide length between 7 and 144 amino acids, 20 ppm for precursor mass tolerance, 0.05 Da for fragment mass tolerance, and maximum identification false discovery rate (FDR) of 1% for both peptide-spectrum match and PP levels.

For GP analysis, all raw data were analyzed with an extra Byonic node in Proteome Discoverer 2.5 software. The carbamidomethyl of cysteine was set as the static modification, while protein N-terminal acetylation, deamination at asparagine and glutamine, and oxidation on methionine were set as the dynamic modifications. The glycan database for Byonic search included 132 human N-glycans, 15 human immunoglobulin M N-glycans, 182 human N-glycans without multiple fucosylation, 59 common biantennary N-glycans, and 57 human plasma N-glycans. The GP identification was performed using the following parameters: maximum of two missed cleavages, maximum of four modifications, maximum of 1 glycan, peptide length between 6 and 144 amino acids, 20 ppm for precursor mass tolerance, 0.05 Da for fragment mass tolerance, and maximum identification FDR of 1% at both peptide-spectrum match and GP levels.

### Data Analysis

The correlation matrix of the four-cell dataset was plotted by the abundance of GPs and PPs expression. The differentially expressed GPs and PPs were statistically analyzed by Student *t* test with *p* value <0.05 by two-tailed *t* test. The significant GPs and PPs were shown in a heat map plotted by Perseus software (v2.0.5.0, https://maxquant.org/perseus/). These differential peptides were further conducted in pathway analysis and protein–protein interaction network analysis. The functional annotation of proteins with differential peptides was performed by Database for Annotation, Visualization, and Integrated Discovery (DAVID, https://david.ncifcrf.gov/) with Kyoto Encyclopedia of Genes and Genomes (KEGG) pathway database, where the significant annotation would be enriched (FDR < 0.1) for cell model comparison. The protein–protein interaction network was constructed by STRING (v10.0, https://string-db.org/). The confidence cutoff for showing interaction links was set to medium (0.400), where all items of the various evidence types in STRING were selected, including experiments, databases, and coexpression, and contributed to the network construction.

## Results

### Experimental Design of Fe-ZIC-cHILIC Tandem Tip for Simultaneous Enrichment of GPs and PPs

**Fig. 1:**
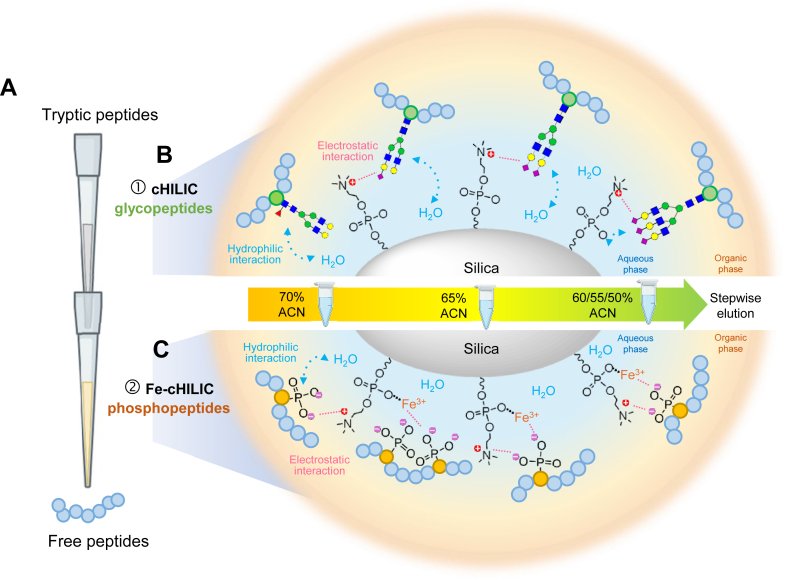
**Principle and enrichment processes of Fe-cHILIC tandem tip for enrichment of glycopeptide and phosphopeptides.***A*, the tandem tip consisted of two individual StageTip. Both tips were packed with ZIC-cHILIC material, while the bottom tip was charged with additional Fe ions, freshly prepared before packing. Peptides from cell lysate were loaded to the tandem tip, where the *top* and *bottom* tip captured intact glycopeptides and phosphopeptides respectively. The free peptides were eluted after sample loading and washing processes. *B*, after capturing glycopeptides and phosphopeptides, the glycopeptides are eluted by either single step or stepwise elution into three fractions: 70% ACN, 65% ACN, and integrated 60%-55%-50% ACN, where the last three fractions were combined into one fraction. *C*, Phosphopeptides are also eluted by stepwise elution into three fractions. ACN, acetonitrile; ZIC-cHILIC, zwitterionic hydrophilic interaction chromatography with the exposed choline group.

Most published studies employed multi-step processes that integrate various materials for either separate or sequential enrichment of GPs and PPs. Under materials of various properties and binding affinity, these approaches may require different loading buffers and elution conditions *via* multiple sample transfer and desalting steps, which may be prone to sample loss, reduced reproducibility, and require large sample input. In this study, we demonstrated a one-material tandem-tip-based strategy, ZIC-cHILIC, to achieve simultaneous enrichment for both intact GPs and PPs (Fig. 1*A*). After the tryptic peptides pass through the tandem tip, the GPs are retained at the first ZIC-cHILIC layer, and unbound peptides pass through the second Fe-ZIC-cHILIC layer to capture PPs. The principle is summarized in the next paragraph.

Compared to the hydrophilic interaction for affinity with GPs in traditional HILIC, we previously demonstrated that ZIC-cHILIC strategy can provide good capture efficiency and high specificity of intact GPs due to the enhanced affinity between exposed positively charged choline group and the negatively charged glycan (Fig. 1*B*) (20). Such an effect is more evident on the significantly enhanced detection percentage and high specificity for SGPs who carry negatively charged sialic acid. Because PPs also carry negative charge, the exposed positive choline group may also attract PPs *via* electrostatic interaction (Fig. 1*C*). To implement ZIC-cHILIC for high performance enrichment of PPs while differentiating its affinity toward the GPs, we further hypothesize that metal ions, such as Fe^3+^ ions, *via* coordination bonding on ZIC-cHILIC may create an unique and stronger affinity for PPs through strong electrostatic interaction (Fig. 1*C*). Thus, the top layer of ZIC-cHILIC layer and bottom layer of Fe^3+^ ions decorated ZIC-cHILIC can independently capture intact GPs and PPs, respectively. Additionally, elution by using different percentages of ACN allows effective fractionation to reduce the complexity and enhance the coverage. This advancement in this approach can collocate as a tandem StageTip. For the whole process, the tandem StageTip reduces multisteps of sample transfer, peptide cleanup, and buffer exchange before LC-MS/MS analysis.

### Optimization of Enrichment Performance of ZIC-cHILIC StageTip for GPs and PPs

**Fig. 2:**
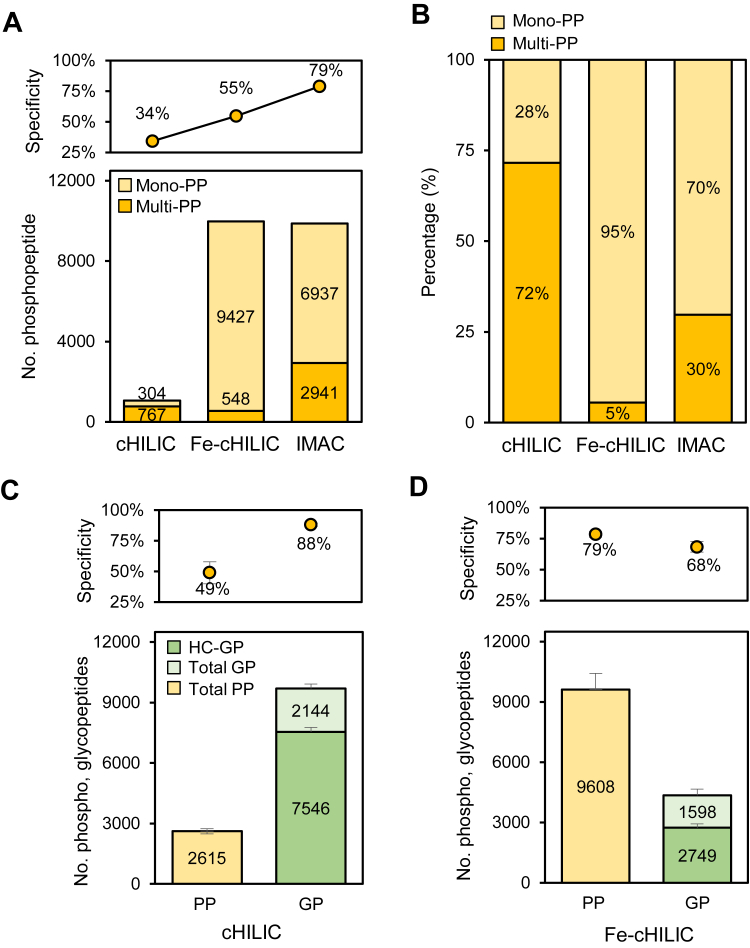
**Comparison of enrichment performance for phosphopeptide and glycopeptides by cHILIC and Fe-ZIC-cHILIC.** The performance was evaluated by using 200 μg protein digest from PC9 cells. *A*, comparison of the number of phosphopeptides (PPs) and enrichment specificity by cHILIC, Fe-ZIC-cHILIC, and IMAC. *B*, comparison of the percentage of multiphosphosites and monophosphosites in cHILIC, Fe-ZIC-cHILIC, and IMAC. *C*, the cHILIC shows preference to enrich significantly more glycopeptides (GPs) compared to PP. HC-GP stands for high-confident glycopeptide with the criteria: Byonic Score >150, PEP2D < 0.01, and LogProb >2. *D*, the Fe-ZIC-cHILIC has the preference to enrich PP compared to GP. Fe-ZIC-cHILIC, Fe-zwitterionic hydrophilic interaction chromatography with the exposed choline group; IMAC, immobilized metal affinity chromatography.

The pH and concentration of acid in the loading condition is critical to affect the degree of negative charge on GPs and PPs as well as the acidic amino acids which cause nonspecific enrichment (7), thus critically controlling the selectivity of GPs and PPs using ZIC-cHILIC material. The performance of PPs enrichment by ZIC-cHILIC material was evaluated by using 50 μg peptides from PC9 cell lysate under 0.1%, 0.5%, and 1% FA or TFA in 80% ACN. Higher specificity (36–43%) was obtained in FA than TFA condition (5–39%) (supplemental Fig. S1*A*). A notably number of PPs was found in Fe^3+^ ions–decorated ZIC-cHILIC (Fe-ZIC-cHILIC) at 0.1% TFA (∼3000 PPs) and FA (supplemental Fig. S1*B*). These results showed that proper control of acid can optimize the enrichment recovery and specificity. Additionally, we had evaluated how the Fe^3+^ concentration influenced the enrichment performance of PPs using Fe-ZIC-cHILIC StageTip. The enrichment efficiency was dependent on the amount of doped Fe^3+^, and excess amount may cause lower recovery due to the presence of excess free Fe^3+^ to bind to PPs. The concentration of Fe^3+^ ions was optimized and 50 mM was chosen for optimal recovery and specificity of PPs (supplemental Fig. S2). After optimization of Fe-ZIC-cHILIC, we further compared the performance of PP enrichment in 200 μg peptides using three different StageTips: ZIC-cHILIC coupled with or without Fe^3+^ ions (Fe-ZIC-cHILIC and cHILIC) and the conventional Fe-IMAC strategy (7). The results show that presence of Fe^3+^ (Fe-ZIC-cHILIC, 9975 PPs) significantly outperformed the cHILIC (1071 PPs) and has comparable coverage to the Fe-IMAC strategy (9878 PPs) (Fig. 2*A* and supplemental Tables S1–S3). The Fe-ZIC-cHILIC strategy also provided a notably higher enrichment specificity of PP (55%) than cHILIC (34%). Interestingly, the Fe^3+^ turned the preference of multiphosphopeptides (multi-PPs) in cHILIC (72% multi-PPs) into predominantly monophosphopeptides (mono-PPs) in Fe-ZIC-cHILIC (95% mono-PPs, Fig. 2*B*), while Fe-IMAC captured more multi-PPs (30%, Fig. 2*B*). In addition to the hydrophilic interaction, the result may be attributed to characteristic to the different chelating group structures between Fe-ZIC-cHILIC and Fe-IMAC. Fe^3+^ ions may coordinate with the negatively charged phosphoric group immobilized on the inner layer of the cHILIC structure. As a result, peptides with multiple phosphorylation sites may experience steric hindrance from the outer barrier formed by choline groups during the enrichment process. The result also suggested that the interaction between phosphate and positive choline groups is weaker than that with Fe^3+^ ions. This property also explains why Fe-ZIC-cHILIC offered lower specificity (55%) than that of Fe-IMAC (79%). Therefore, Fe-ZIC-cHILIC significantly improved the specificity of PP enrichment compared to cHILIC and provided a comparable PP coverage with Fe-IMAC strategy (supplemental Fig. S3*A*) that obtained more hydrophobic PPs (supplemental Fig. S3*B*).

Following the optimal enrichment of PPs, we further evaluated the capture efficiency of cHILIC and Fe-ZIC-cHILIC for GPs and PPs using 200 μg samples. Under triplicate enrichments, we found that cHILIC prefers to enrich GPs with high specificity (88%) as well as large coverage of 9690 GPs with 7546 high-confident GPs (HC-GP) (Byonic Score >150, PEP2D < 0.01, and LogProb >2) (Fig. 2*C*), while relatively fewer PPs were identified by cHILIC enrichment. On the other hand, Fe-ZIC-cHILIC enhanced enrichment of significantly more PPs (9608 PPs, 79% specificity), yet has low capture ability and coverage of 4347 GPs (2749 highly confident GPs) and 68% specificity (Fig. 2*D*). This performance indicates that cHILIC and Fe-ZIC-cHILIC could be complementary for the simultaneous or sequential enrichment of GPs and PPs. We further explored the sensitivity of N-glycoproteome and phosphoproteome by cHILIC and Fe-ZIC-cHILIC StageTip approach. In case, without protein immunoprecipitation, the approach identified important glycosylation and phosphorylation on druggable protein, such as 10 of 12 N-glycosylation sites and driver autophosphorylation sites, such as Y1172 and Y1110 of EGFR protein, the druggable target in lung cancer (supplemental Fig. S4). In addition, most of the glycoproteins and phosphoproteins involved in the NSCLC pathway were identified (supplemental Fig. S5). The results showcase the sensitivity of this approach through its high coverage of many oncoproteins and their downstream signaling cascades.

### Iron-Enhanced ZIC-cHILIC Tandem Tip Achieved Simultaneous Enrichment of GPs and PPs

We next designed a tandem StageTip integrating the two layers of cHILIC and Fe-ZIC-cHILIC (Fig. 1A). We evaluated two configurations of different enrichment order of the two layers to achieve simultaneous enrichment with optimal enrichment performance in identification coverage and recovery of GPs and PPs from the same sample (200 μg peptides).

**Fig. 3:**
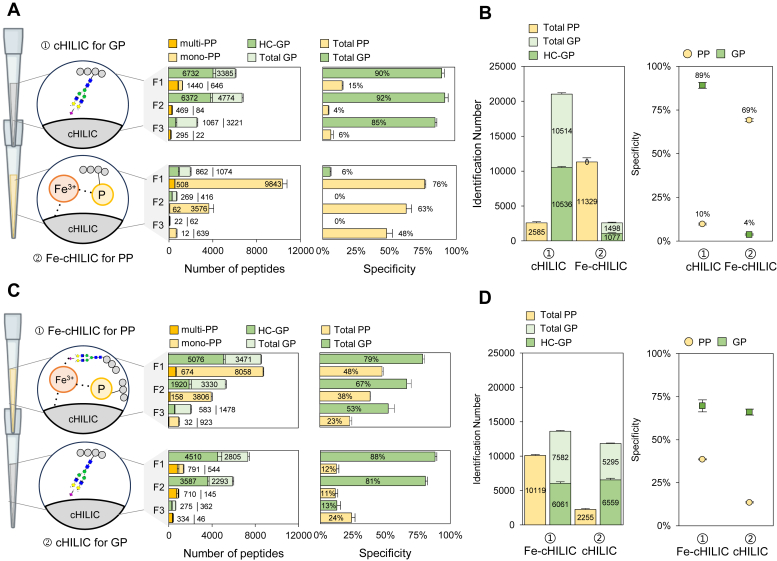
**Evaluation on the order of sequential experiment of glycopeptides and phosphopeptides by Fe-cHILIC tandem tip.***A*, enrichment is performed in the order of ① cHILIC for glycopeptides (GPs) and ② Fe-ZIC-cHILIC for phosphopeptides (PPs). Performance is shown on the number of identified GPs (total GP), high-confident GPs (HC-GPs), mono-PPs, multi-PPs, and enrichment specificity of PPs and GPs in the three fractions: F1 (70% ACN), F2 (65% ACN), F3 (combination of 60%-55%-50% of ACN/H_2_O solutions). *B*, summary of total number of peptides and enrichment specificity (%) by combining three fractions. *C*, enrichment is performed in the order of ① Fe-ZIC-cHILIC for PPs and ② cHILIC for GPs. Performance is shown on the number of identified GPs (total GP), HC-GPs, mono-PPs, multi-PPs, and enrichment specificity of PPs and GPs in the three fractions. *D*, summary of total number of peptides and enrichment specificity (%) by combining three fractions. Fe-ZIC-cHILIC, Fe-zwitterionic hydrophilic interaction chromatography with the exposed choline group; ACN, acetonitrile.

The first test involves Tip 1 of cHILIC for GPs, followed by Tip 2 of Fe-ZIC-cHILIC for PP enrichment (Fig. 3*A*, upper panel). To further improve the identification coverage, a simple three-step fractionation was also performed. Under this setup, the first cHILIC StageTip shows >6300 HC-GPs for both fraction 1 and 2 and high specificity (85–92%) in all three fractions (Fig. 3*A*, upper panel, supplemental Table S4). As expected, the first cHILIC StageTip designed for GP enrichment did not have good performance for PP; only a low number of PPs with low enrichment (4–15%) were achieved (Fig. 3A, upper panel). Nevertheless, the second Fe-ZIC-cHILIC tip shows great performance for PP enrichment, mainly on the first two fractions which enriched 10,351 and 3638 PPs with specificity of 76% and 63%, respectively (Fig. 3*A*, lower panel, supplemental Table S5). The enrichment has preference on the mono-PPs, 70 to 85% in fraction 1 and 2. Moreover, almost no GPs were coeluted in the Fe-ZIC-cHILIC eluates. By integrating results from all three fractions, it is clear that the top cHILIC preferentially captures GPs (10,536 HC-GPs), while the bottom Fe-ZIC-cHILIC shows preference in PPs (11,329 PPs) (Fig. 3*B*). Among the GPs identified by the top cHILIC, it is interesting to note a significant proportion of 4285 highly confident SGPs (supplemental Fig. S6, *A* and *B*). Overall, the top cHILIC and bottom Fe-ZIC-cHILIC configuration demonstrated high specificity for GPs (89%) and PPs (69%) (Fig. 3*B*).

When the order of tandem StageTip assembly is reversed, Tip 1 for Fe-ZIC-cHILIC and Tip 2 for cHILIC materials, the overall enrichment performance remains at a similar level for PPs, while the enrichment coverage significantly reduces for GPs. The top Fe-ZIC-cHILIC tip enriched 8732 and 3964 PPs with specificities of 48% and 38% in the first two fractions, respectively (Fig. 3*C*, upper panel, supplemental Table S6). However, Fe-ZIC-cHILIC also captured a significant portion of 5076 HC-GPs, accompanied by 1634 SGPs in the first fraction (supplemental Fig. S6*C*). As expected, the subsequent Tip 2 (cHILIC phase) has preference for GP, yet lower numbers, 4,510, 3,587, and 275 HC-GPs, were captured with specificities of 88%, 81%, and 13% in three fractions, respectively (Fig. 3*C*, lower panel, supplemental Table S7). Combining results from all three fractions, the two cHILIC and Fe-ZIC-cHILIC did not show distinct preference toward GPs and SGPs (Fig. 3*D* and supplemental Fig. S6*D*). Furthermore, the overall performance shows lower identification number and coverage in both GPs and PPs than the results from the assembly order of cHILIC followed by Fe-ZIC-cHILIC. The peptide alignment and quantitation result revealed that cHILIC prefers to capture GPs and Fe-ZIC-cHILIC favors PPs (supplemental Fig. S7, *A* and *B*). However, the enrichment efficiency of Fe-ZIC-cHILIC for PPs may be affected by the presence of GPs, especially the SGPs.

We previously reported that different metal ions, such as Fe^3+^, Ga^3+^, and Ti^4+^, coordinated to IMAC enriched different types of PPs due to their different strength of positive charges (30). Thus, we have compared the performance of PP enrichment *via* Ga-ZIC-cHILIC and Fe-ZIC-cHILIC. supplemental Fig. S8*A* summarizes their identification coverage and PP characteristics. When Fe was first doped into cHILIC followed by Ga, 6033 and 4112 PPs were identified in Fe-ZIC-cHILIC and Ga-ZIC-cHILIC, respectively. Consistent with the findings of our previous study (30), Ga showed a preference for capturing multi-PPs (17%), whereas only 6% is multi-PP in Fe-ZIC-cHILIC, consistent with only 5% multi-PPs among all enriched PPs in Fe-ZIC-cHILIC only (Fig. 2*A*). Common PPs (2467 PPs, 44.2%) indicates complementary nature of Ga- and Fe-ZIC-cHILIC (supplemental Fig. S8*B*). The GRAVY score distribution, a feature indicative of PP hydrophobicity, indicated that Fe-ZIC-cHILIC tended to be more positive compared to Ga-ZIC-cHILIC. When the doping order was reversed, slightly lower identification results, 1683 and 6416 PPs, were observed in Ga-ZIC-cHILIC and Fe-ZIC-cHILIC, respectively (supplemental Fig. S8*C*). In this case, Ga captured significantly more multi-PP (26%), while Fe maintained a similar percentage of multi-PP (7%) and more hydrophobic PPs (supplemental Fig. S8*D*).

The above results show that different metal ions possess complementary enrichment performance toward comprehensive coverage. Thus, we have also tested dual-metal ions doped in ZIC-cHILIC material, which is composed of both Fe and Ga doped in the same StageTip. supplemental Fig. S9*A* shows the comparison of enrichment performance by packing order of Ga ions, followed by Fe ions (Ga-ZIC-cHILIC>Fe-ZIC-cHILIC), or by reverse packing order (Fe-ZIC-cHILIC>Ga-ZIC-cHILIC). The result shows that the performance of Ga-ZIC-cHILIC>Fe-ZIC-cHILIC (6969 PPs) slightly increases the PP identification coverage when compared with Fe-ZIC-cHILIC only (6033 PPs) or Ga-ZIC-cHILIC only (1683 PPs). Compared to the Fe-ZIC-cHILIC, the percentage of multi-PPs increased from 6% to 10%. When packing order is reversed (Fe-ZIC-cHILIC > Ga-ZIC-cHILIC), 5222 PPs were identified and the percentage of multi-PPs remaining the same (supplemental Fig. S9*B*). Both methods show high common PPs (73.4%) (supplemental Fig. S9*C*) and high similarity in the PP hydrophobicity (supplemental Fig. S9*D*). Thus, we choose the use of a single metal ion, Fe-ZIC-cHILIC, for the simplicity and efficiency.

**Fig. 4:**
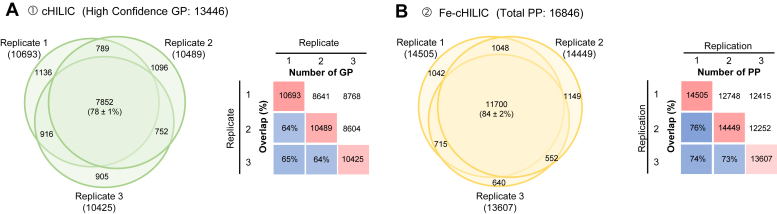
**Evaluation of reproducibility by triplicate sequential enrichment in the order of cHILIC for glycopeptides enrichment and Fe-ZIC-cHILIC for phosphopeptides.***A*, Venn diagram showing the number and percentage of overlapping high-confident glycopeptides (HC-GPs) from triplicate experiments of cHILIC tip. Common HC-GPs in three replicates account for 78 ± 1% (7852 HC-GPs). *B*, Venn diagram showing the number and percentage of overlapping phosphopeptides from triplicate experiments of Fe-ZIC-cHILIC tip. Common PPs in three replicates account for 84 ± 2% (11,700 PPs). Fe-ZIC-cHILIC, Fe-zwitterionic hydrophilic interaction chromatography with the exposed choline group.

Based on the above results, we further evaluated the reproducibility through triplicate experiments, specifically the consistency in peptide sequences, N-glycosites, modifications, and glycan compositions in GPs, as well as peptide sequences and phosphosites in PPs. The results show higher common identification in PPs (11,700, 84 ± 2%) than the commonly identified HC-GPs (7,852, 78 ± 1%) in the tandem tips (cHILIC→Fe-ZIC-cHILIC), respectively (Fig. 4, *A* and *B*). Even reversing the order of the tandem tips, the reproducibility remained similar level for the commonly identified PPs (11,840, 84 ± 1%) and HC-GPs (4,520, 75 ± 3%) in the Fe-ZIC-cHILIC→cHILIC tandem tips, respectively (supplemental Fig. S10, *A* and *B*). In summary, these results show that cHILIC-based tandem tips, in the order of cHILIC→Fe-ZIC-cHILIC, offer simultaneous glycoproteomics and phosphoproteomics profiling with high specificity and good reproducibility.

### Application in Different EGFR-Mutant NSCLC Cells

**Fig. 5:**
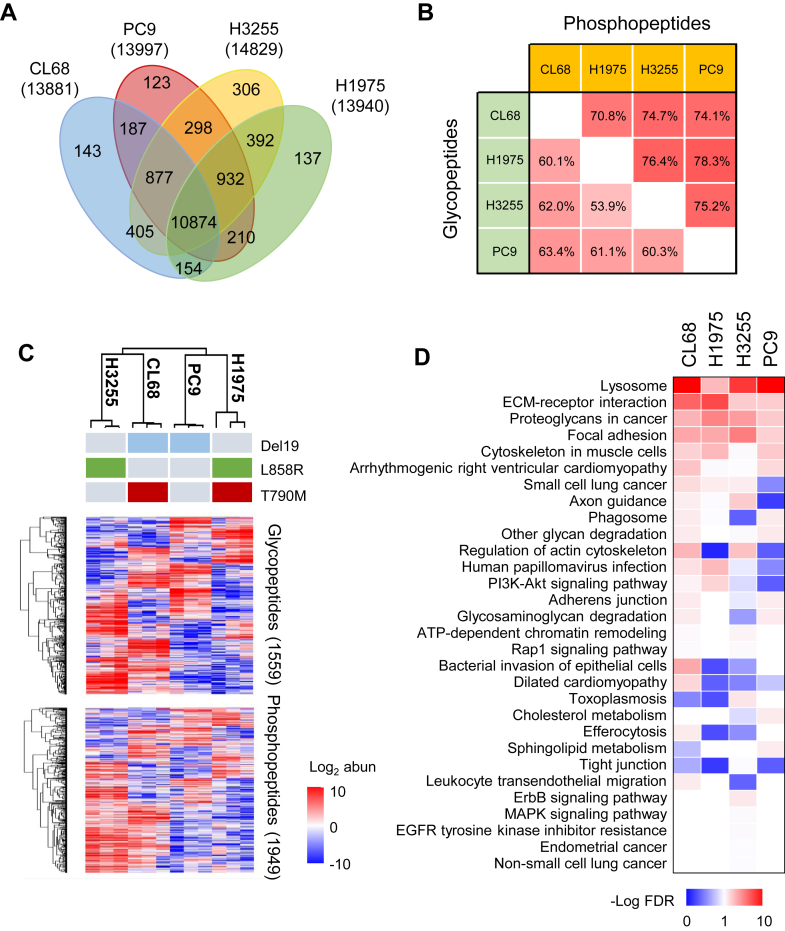
**N-glycoproteomic and phosphoproteomic profile in four different EGFR subtypes of NSCLC cells.***A*, Venn diagram showing the number of overlapping modified peptides, including high-confident N-glycopeptides (GPs) and phosphopeptides from four cells by using tandem cHILIC-Fe-ZIC-cHILIC StageTip. High-confident GPs were selected by Byonic Score >150, PEP2D < 0.01, and LogProb >2. *B*, correlation between four cells was evaluated by GPs and PPs. *C*, heat map presents the differential profile from N-glycoproteome and phosphoproteome. *D*, the differential GPs and PPs were further analyzed by DAVID functional annotation tool. The significant KEGG pathways (-Log FDR > 1) were compared in these four cells. Fe-ZIC-cHILIC, Fe-zwitterionic hydrophilic interaction chromatography with the exposed choline group; FDR, false discovery rate; KEGG, Kyoto Encyclopedia of Genes and Genomes; DAVID, Database for Annotation, Visualization, and Integrated Discovery; NSCLC, non–small cell lung cancer.

Lung cancer remains the leading cause of cancer-related deaths globally. EGFR mutations, such as Del19 and L858R, are common in NSCLC and have been effectively targeted by EGFR TKIs (31). However, resistance to TKIs typically develops within 9 to 15 months in about 70% of patients (32), with the EGFR T790M mutation accounting for 60% of cases (33). Despite this, the mechanisms driving acquired resistance, including the roles of glycosylation and phosphorylation, remain unclear and warrant further investigation. To further demonstrate the applicability of the developed tandem tip to explore the interplay between N-glycosylation and phosphorylation of the real world samples, we applied the approach to quantitatively compare four different NSCLC cell lines exhibiting either sensitivity or resistance to the TKI targeted EGFR. The comparison includes two sets of cell lines harboring common onco-driver mutations—Del19 or L858R—with (CL68 cell-Del19 and H1975 cell-L858R) or without T790M mutation (PC9 cell-Del19 and H3255 cell-L858R) on EGFR, presenting resistant or sensitive response for TKI therapy. Triplicate experiments were performed for these 4 cell lines (200 μg peptides) by the tandem tip strategy (cHILIC→Fe-ZIC-cHILIC StageTips) with three stepwise elutions, followed by LC-MS/MS analysis and label free quantitation. Similar numbers of GPs, SGPs, and PPs were quantified in the four cell lines and the total unique modified peptides in each cell were an average of 14,162 ± 447 (supplemental Fig. S11*A*). Among the total of 16,279 unique modified peptides which integrate 6867 unique GPs from 383 glycoproteins and 9412 unique PPs from 1132 phosphoproteins, 10,874 peptides were identified with glycosylation or phosphorylation and can be quantified in all of these four cells (Fig. 5*A*). The Venn diagram shows that the majority of these proteins are commonly present in all 4 cell lines; only less than 2% of GPs and PPs were uniquely identified in one type of EGFR mutant cells. Moreover, the abundance of PPs between the four cell lines show higher correlation (71–78%) compared to that of N-GPs (54–63%), likely due to the complex variation of the glycan structures in the same glycosylation sites (Fig. 5*B*). To understand the potential regulators through glycosylation and phosphorylation, we found 88 proteins containing both of two modifications (supplemental Fig. S11*B*). To identify the potential regulators through interplay of glycosylation and phosphorylation, we found that 23 proteins are annotated as plasma membrane proteins from Gene Ontology resource and many of them are FDA-approved drug targets, such as EGFR, ERBB2, MET, and integrin family members, suggesting our result may provide enrichment of cell surface glycosylation and intracellular phosphorylation cascades to study their relationship.

To further illuminate the alterations of these two modifications associated with either EGFR mutation status or TKI resistance, the expression level of unique GPs and PPs were calculated by statistics analysis using student *t* test (*p* < 0.05). These significantly differential peptides, including 1559 GPs and 1949 PPs, were selected and categorized by hierarchical clustering (Fig. 5*C*). Both the differential N-GPs and PPs have the similar trend to cluster the H3255 and CL68, PC9 and H1975 cell lines. Surprisingly, such clustering results indicate that either EGFR mutation or TKI resistance did not affect their clustering similarity in the 4 cell lines; both glycosylation and phosphorylation profiles show the same clustering results with distinction between the 4 cell lines. The functional annotation of signaling pathways for these functional annotations in signaling pathways for the proteins with these discriminative GPs and PPs were mapped to the KEGG database (34). The top enriched KEGG pathways include lysosome, extracellular matrix–receptor interaction, proteoglycan in cancer, focal adhesion, suggesting potential involvement of membrane remodeling, organization, and lysosomal degradation (FDR <0.1, Fig. 5*D*). Most interestingly, two pathways—human papillomavirus infection and PI3K–Akt signaling pathway—were uniquely enriched only in the TKI-resistant cells, CL68 and H1975 harboring acquired EGFR-T790M mutation.

**Fig. 6:**
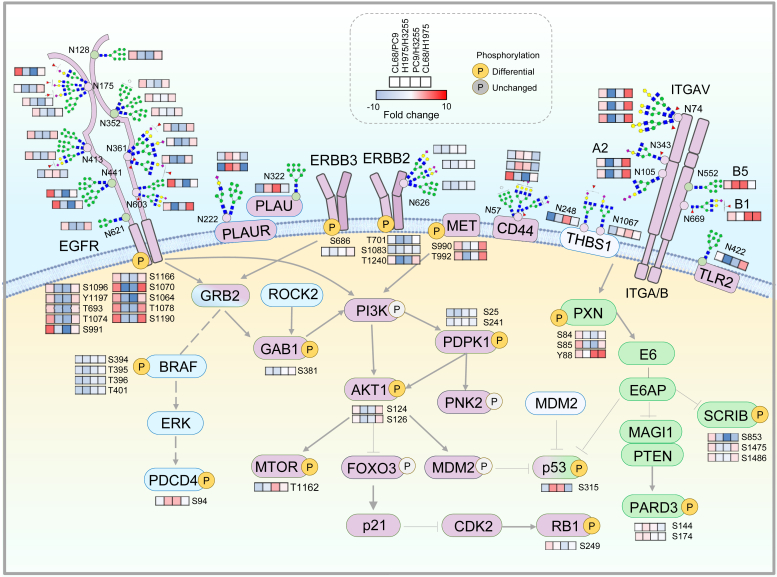
**Illustration of N-glycoproteomic and phosphoproteomic profile in TKI-resistant NSCLC cells.** The differentially expressed targets, including N-glycosylation and phosphorylation, were selected from plasma membrane receptors, ERBB pathway, PI3K–Akt pathway, mTOR pathway, and proteoglycan in cancer. NSCLC, non–small cell lung cancer; TKI, tyrosine kinase inhibitor.

The NSCLC pathway and its subaxis are the major signaling pathways for patients with EGFR activation mutations (Del19 and L858R). The tandem-tip strategy identified differential site-specific glycosylation and phosphorylation from the highly relevant pathways, such as NSCLC, ErbB signaling pathway, and most well-known resistance-related EGFR TKI resistance pathway (Fig. 5*D*). We observed highest level of the known EGFR mutant–induced autophosphorylation sites, Y1197, in CL68 cells compared to H3255, PC9, and H1975 cells, indicating that the phosphosite associated with Del19 accompanied T790M on EGFR (supplemental Fig. S12). The EGFR-Y1092 site was also detected at higher levels in CL68 and H1975 cells, which is consistent with previous reports on its expression in EGFR-T790M cells and association with gefitinib resistance (35). The result revealed that our approach provides good sensitivity in phosphoproteomic profiling to detect the EGFR activating site as well as the TKI-resistant sites. From an N-glycoproteomic view, we also observed a significant number of differential site-specific glycosylation on EGFR in the resistant cell lines. There are 10 biantennary and triantennary fucosyl-sialo-glycans at N603 on EGFR with highest level in CL68 cells (Fig. 6 and supplemental Fig. S12). The result provided a new insight at glycosylation level and the site-specific alteration in the TKI-resistant T790M mutant NSCLC cells.

In the overall N-glycoproteomic and phosphoproteomic profile, a total of 1427 proteins have concomitant changes in the glycosylation and phosphorylation. The majority of differential targets with higher levels in T790M cells were modifications on the EGFR and integrin protein family (supplemental Table S8). For instance, dramatic alterations on the glycan structures were observed in some proteins in the T790M-bearing NSCLC cells. For the most widely used targeted therapy against EGFR, our work identified 48 EGFR GPs among 11 out of 12 sites and 11 PPs (supplemental Table S8). One sialo-fucosyl-biantennary glycan at EGFR-N361 has the highest level in H1975 cells. Similar trend was observed in the detection of a sialo-fucosyl-monoantennary glycan at N74 on ITGAV, and a few sialo-fucosyl-biantennary and triantennary glycans at N343 on ITGA2 (Fig. 6). Noticeably, the phosphorylation levels at Y1197 and T693 on EGFR were observed with highly positive correlation with its glyco-profile expression in both TKI-resistant NSCLC cells (CL68 and H1975 cells). Accompanying the glycosylation changes on EGFR in the resistant cell lines, the PI3K–Akt pathway, the EGFR downstream cascades, is one of the major pathways with presence of many differential phosphosites, such as T1240 on ERBB2, S990/T992 on MET, S124/S126 on AKT1, observed in CL68-resistant cells, while these phosphosites have lower levels in TKI-sensitivity PC9 cells. Interestingly, these phosphosites also presented higher levels in CL68 cells with Del19 than H1975 cells with L858R. These concomitant changes on the elevated glycosylation pattern and autophosphorylation sites on EGFR and its downstream PI3K and MAPK pathway suggest that long-term treatment with EGFR-TKI likely induced the dysregulation of these extracellular glycosylation and intracellular phosphorylation on mutated EGFR, which may eventually affect the TKI responses. Our quantitative glycoproteomic and phosphoproteomic analysis not only illuminated the full landscape and association between cell surface (sialo-)glycosylation and the intracellular phosphorylation cascade but also identified the major alterations in the TKI-resistant cells.

## Discussions

In this study, we have demonstrated for the first time that the ZIC-cHILIC material can be successfully applied to simultaneously enrich both PPs and GPs with high specificity. The ZIC-cHILIC contains phosphoryl group in the internal side and exposes the positive choline group at terminus, allowing the effective capture for GPs, especially negatively charged SGPs, through hydrophilic interaction and strong electrostatic attraction (20). Addition of metal ions, such as Fe^3+^, to coordinate with negatively charged phosphoryl group on the inner layer of the cHILIC structure is a new concept for ZIC-cHILIC–based material; the coordinated Fe^3+^ ions offer strong electrostatic binding affinity toward PPs, similar to the principle in the popular tool Fe-IMAC. Thus, this approach demonstrated good specificity in both GPs (89%) and PPs (69%).

Compared to previous work, our strategy offers several advantages. Different from traditional enrichment methods, our strategy utilizes one-material tandem tips and design a single buffer for material activation, peptide incubation, and simultaneous enrichment of two PTMs by the cHILIC—Fe-ZIC-cHILIC tandem StageTip. By packing ZIC-cHILIC–based silica beads into a tandem tip, ZIC-cHILIC and charge Fe^3+^ ion to tune the selectivity toward PPs, this strategy achieves a simple and efficient workflow for simultaneous profiling of N-glycoproteomics and phosphoproteomics. These ZIC-cHILIC material and the StageTips are commercially accessible that can be easily packed into other formats such as microtips and agarose gel for the proteomic community. By integrating two StageTip into a single tip format, our goal was to address the challenges of labor-intensive procedures, and the risk of sample loss commonly associated with traditional multistep tube transfer processes during the enrichment. This integrated approach eliminates the need for multiple sample transfer and collection steps, additional peptide desalting, and buffer exchanges, resulting in a significantly streamlined workflow. Another key innovation in our strategy is the functionality of stepwise elution and fractionation, which enhance more than 4-fold coverage in the number of GPs compared to single-step elution in our previous work (20). In terms of sample amount and identification coverage, this study demonstrated competitive sensitivity and profiling depth by identification of more than 10,000 GPs and PPs from 200 μg cell lysate, which is 3-fold than previous work using fractionation strategy to analyze 500 μg mouse lung tissue (28). Compared to direct enrichment methods without fractionation which offer coverage of around one to a few thousands of GPs and PPs, our approach had even higher identification coverage (24, 27). Chen *et al*. reported the deepest identification of 8764 N-GPs and 4890 PPs from 200 μg of small extracellular vesicle peptides using a TiO_2_ and HILIC-based strategy (36), while our approach achieves greater coverage with faster processing.

This study provides a large-scale glycoproteomic and phosphoproteomic profile to depict the landscape of dual modifications in sensitive and resistant NSCLC cells. Differentially expressed glycoprofile with diversified glycan structures also distinguished EGFR mutant types and TKI resistance in NSCLC cells in the presence or absence of secondary T790M mutation. This dual PTMomic landscape reveals the complex relationship between driver gene mutation, protein glycosylation and phosphorylation. Extracellular glycosylations on plasma membrane proteins may act as a barrier to intervene drug therapy in cancer cells. Some studies have reported that higher degree of sialylation and phosphorylation interactively regulate EGFR dimerization and activation in TKI-resistant EGFR mutant cells and sialidase can increase EGFR dimerization upon EGF treatment in lung cancer cells (2). Disturbing glycosylation profile on cell surface impacts the drug response and targeting N-glycosylation may improve cancer chemotherapy and reduce drug resistance (37). Consistent with the literature that increased level of poly-LacNAc chain and β1,6-GlcNAc bearing N-glycans implicate in drug resistance, our dataset provides a site-specific evidence that the glycan HexNAc(6)Hex(7)Fuc(2)NeuAc(1) on ITGA2-N343 has higher expression level in the two TKI-resistant cells than the sensitive cells of the same EGFR mutation. In the TKI-resistant CL68 and H1975 cells, most prominent alterations in glycosylation and phosphorylation were observed in EGFR itself, the downstream PI3K–Akt signaling pathway and integrin family in resistant cells. The result may link with the key regulator ST6GalT-1, which has been reported to enhance migration and invasion of tumor cells, and the enzyme activates PI3K–Akt signaling pathway by targeting integrins and potentially some RTKs (38, 39). On the other hand, abnormal α2,6-sialylation of β1 integrin subunit, which was also observed in this study, has been reported to enhance its activity and subsequent cell migration by improving extracellular matrix/cytoskeleton interactions (40). In summary, the established PTMomics may provide a site-specific knowledgebase to further study the potential roles of N-glycoproteome, phosphorylation cascade, and their interplay underlying the PTM-induced mechanism underlying the TKI resistance.

Aside from this study, the EGFR glycosylation and phosphorylation have been characterized in other cancer types. By tandem mass tag 11-plex strategy, Liu *et al.* identified glycosylated N352 and N603 sites, both in proximity of the EGFR mutation sites, which was found to have positive associations with the Y316 phosphorylation site in patients with EGFR mutation (41). These results suggest the potential cross-talk between PTMs and somatic mutations underlying the biology of driver events. By tandem mass tag 10-plex strategy, Lih *et al.* also discovered the interactions specifically in clear cell renal cell carcinoma (42). The linear regression was found between the specific EGFR glycosylation site (EGFR-N352, 2 HexNAc, and 5 Hexose, N2H5) and its phosphorylation site (EGFR-S1018). The observed association suggests that the N352 glycosylation mediate S1018 phosphorylation, which subsequently affecting EGFR stabilization (42). These works showed that EGFR glycosylation and phosphorylation coregulated the development of two different cancer types. In this study, our identification coverage included the above-mentioned glycosylation sites, N352 (N2H5, N2H6, N2H7, N2H8, and N2H10), N603 (N2H5, N2H7, N4H5F1, N3H5F1S1, N4H5F1S1, N4H5F1S2, N4H5F2S1, N4H5F3S1, N4H6F0S2, N5H5F0S1, N5H6F1S1, N5H6F1S2, N5H6F3S1), as well as the most critical EGFR mutant–induced autophosphorylation sites, T693 and Y1197. Quantitative comparison reveals that Y1197 and T693 on EGFR were observed with highly positive correlation with its glyco-profile, especially the sialo-fucosyl-biantennary glycan (N5H5F1S1 on N413, N6H3F1S1 on N444, N4H5F1S1 on N603) in both TKI-resistant NSCLC cells. The result revealed that our approach provides good sensitivity in phosphoproteomic profiling to detect the EGFR activating site as well as the TKI-resistant sites. Taken together, the EGFR cross-talk between glycosylation and phosphorylation is likely a pan-cancer phenomenon in different cancer types, which required further investigations to explore its detailed regulatory mechanism.

## Conclusions

In summary, our one-material tandem tip strategy offers a powerful tool for investigating the complex relationship of N-glycosylation and phosphorylation and advancing our understanding of their profiles related to TKI resistance. We demonstrate that ZIC-cHILIC offers high enrichment performance for GPs and PPs, and the Fe-ZIC-cHILIC has higher specificity for PPs through the stronger Fe-coordinated electrostatic affinity. Different with previous strategies, this one-material ZIC-cHILIC tandem tip possesses simultaneous enrichment of N-glycoproteome and phosphoproteome and supports stepwise fractionation to enhance the coverage of GPs and PPs in one sample. This study demonstrated deep PTM profiling and good reproducibility. The application to TKI-resistant NSCLC model study reveals, for the first time, on how EGFR mutation altered its glycosylation, phosphorylation, and downstream phosphorylation cascade, providing valuable insights into TKI resistance and potential therapeutic targets that warrant further functional validation. Unraveling dual PTM interplay paves the way for in-depth investigations on the link between cell surface glycoprofile and intracellular signaling networks. With the commercially accessible of the ZIC-cHILIC material and StageTip, our strategy can be easily adapted for different sample types in the community.

## Data Availability

The mass spectrometry proteomics data have been deposited to the ProteomeXchange Consortium *via* the PRIDE partner repository (43) with the dataset identifier PXD056077.

## Supplemental data

This article contains supplemental data.

## Conflict of interest

The authors declare no competing interest.

## References

[1] Beltrao P., Albanèse V., Kenner L.R., Swaney D.L., Burlingame A., Villén J. (2012). Systematic functional prioritization of protein posttranslational modifications. Cell.

[2] Liu Y.-C., Yen H.-Y., Chen C.-Y., Chen C.-H., Cheng P.-F., Juan Y.-H. (2011). Sialylation and fucosylation of epidermal growth factor receptor suppress its dimerization and activation in lung cancer cells. Proc. Natl. Acad. Sci. U. S. A..

[3] Mereiter S., Balmaña M., Campos D., Gomes J., Reis C.A. (2019). Glycosylation in the Era of cancer-targeted therapy: where are we heading?. Cancer Cell.

[4] Kaszuba K., Grzybek M., Orłowski A., Danne R., Róg T., Simons K. (2015). N-Glycosylation as determinant of epidermal growth factor receptor conformation in membranes. Proc. Natl. Acad. Sci. U. S. A..

[5] Mereiter S., Magalhães A., Adamczyk B., Jin C., Almeida A., Drici L. (2016). Glycomic analysis of gastric carcinoma cells discloses glycans as modulators of RON receptor tyrosine kinase activation in cancer. Biochim. Biophys. Acta Gen. Subj..

[6] Singh V., Ram M., Kumar R., Prasad R., Roy B.K., Singh K.K. (2017). Phosphorylation: implications in cancer. Protein J..

[7] Tsai C.-F., Wang Y.-T., Chen Y.-R., Lai C.-Y., Lin P.-Y., Pan K.-T. (2008). Immobilized metal affinity chromatography revisited: pH/acid control toward high selectivity in phosphoproteomics. J. Proteome Res..

[8] Ruprecht B., Koch H., Medard G., Mundt M., Kuster B., Lemeer S. (2015). Comprehensive and reproducible phosphopeptide enrichment using iron immobilized metal ion affinity chromatography (Fe-IMAC) columns. Mol. Cell Proteomics.

[9] Tape C.J., Worboys J.D., Sinclair J., Gourlay R., Vogt J., McMahon K.M. (2014). Reproducible automated phosphopeptide enrichment using magnetic TiO2 and Ti-IMAC. Anal. Chem..

[10] Wolschin F., Wienkoop S., Weckwerth W. (2005). Enrichment of phosphorylated proteins and peptides from complex mixtures using metal oxide/hydroxide affinity chromatography (MOAC). Proteomics.

[11] Hogrebe A., von Stechow L., Bekker-Jensen D.B., Weinert B.T., Kelstrup C.D., Olsen J.V. (2018). Benchmarking common quantification strategies for large-scale phosphoproteomics. Nat. Commun..

[12] Kitata R.B., Choong W.-K., Tsai C.-F., Lin P.-Y., Chen B.-S., Chang Y.-C. (2021). A data-independent acquisition-based global phosphoproteomics system enables deep profiling. Nat. Commun..

[13] Muneer G., Chen C.-S., Lee T.-T., Chen B.-Y., Chen Y.-J. (2024). A rapid one-pot workflow for sensitive microscale phosphoproteomics. J. Proteome Res..

[14] Liu M.-Q., Zeng W.-F., Fang P., Cao W.-Q., Liu C., Yan G.-Q. (2017). pGlyco 2.0 enables precision N-glycoproteomics with comprehensive quality control and one-step mass spectrometry for intact glycopeptide identification. Nat. Commun..

[15] Wang Z., Wang J., Sun N., Deng C. (2019). A promising nanoprobe based on hydrophilic interaction liquid chromatography and immobilized metal affinity chromatography for capture of glycopeptides and phosphopeptides. Anal. Chim. Acta.

[16] Xu Y., Wu Z., Zhang L., Lu H., Yang P., Webley P.A. (2009). Highly specific enrichment of glycopeptides using boronic acid-functionalized mesoporous silica. Anal. Chem..

[17] Madera M., Mechref Y., Novotny M.V. (2005). Combining lectin microcolumns with high-resolution separation techniques for enrichment of glycoproteins and glycopeptides. Anal. Chem..

[18] Hashim O.H., Jayapalan J.J., Lee C.-S. (2017). Lectins: an effective tool for screening of potential cancer biomarkers. PeerJ.

[19] Zborníková E., Knejzlík Z., Hauryliuk V., Krásný L., Rejman D. (2019). Analysis of nucleotide pools in bacteria using HPLC-MS in HILIC mode. Talanta.

[20] Chen Y.-J., Yen T.-C., Lin Y.-H., Chen Y.-L., Khoo K.-H., Chen Y.-J. (2021). ZIC-cHILIC-Based StageTip for simultaneous glycopeptide enrichment and fractionation toward large-scale N-Sialoglycoproteomics. Anal. Chem..

[21] Swaney D.L., Beltrao P., Starita L., Guo A., Rush J., Fields S. (2013). Global analysis of phosphorylation and ubiquitylation cross-talk in protein degradation. Nat. Methods.

[22] Mertins P., Qiao J.W., Patel J., Udeshi N.D., Clauser K.R., Mani D.R. (2013). Integrated proteomic analysis of post-translational modifications by serial enrichment. Nat. Methods.

[23] Huang J., Wang F., Ye M., Zou H. (2014). Enrichment and separation techniques for large-scale proteomics analysis of the protein post-translational modifications. J. Chromatogr. A..

[24] Zou X., Jie J., Yang B. (2017). Single-step enrichment of N-glycopeptides and phosphopeptides with novel multifunctional Ti^4+^-immobilized dendritic polyglycerol coated chitosan nanomaterials. Anal. Chem..

[25] Cui Y., Yang K., Tabang D.N., Huang J., Tang W., Li L. (2019). Finding the sweet spot in ERLIC mobile phase for simultaneous enrichment of N-glyco and phosphopeptides. J. Am. Soc. Mass Spectrom..

[26] Zheng H., Jia J., Li Z., Jia Q. (2020). Bifunctional magnetic supramolecular-organic framework: a nanoprobe for simultaneous enrichment of glycosylated and phosphorylated peptides. Anal. Chem..

[27] Zhou X., Zhang H., Wang L., Lv L., Wu R. (2023). Simultaneous enrichment optimization of glycopeptides and phosphopeptides with the highly hydrophilic DZMOF-FDP. Analyst.

[28] Wang D., Huang J., Zhang H., Ma M., Xu M., Cui Y. (2023). ATP-coated dual-Functionalized titanium (IV) IMAC material for simultaneous enrichment and separation of glycopeptides and phosphopeptides. J. Proteome Res..

[29] Chen C.-W., Lin P.-Y., Lai Y.-M., Lin M.-H., Lin S.-Y., Hsu C.-C. (2024). TIMAHAC: streamlined tandem IMAC-HILIC workflow for simultaneous and high-throughput plant phosphoproteomics and N-glycoproteomics. Mol. Cell Proteomics.

[30] Tsai C.-F., Hsu C.-C., Hung J.-N., Wang Y.-T., Choong W.-K., Zeng M.-Y. (2014). Sequential phosphoproteomic enrichment through complementary metal-directed immobilized metal ion affinity chromatography. Anal. Chem..

[31] Wang X., Goldstein D., Crowe P.J., Yang J.-L. (2016). Next-generation EGFR/HER tyrosine kinase inhibitors for the treatment of patients with non-small-cell lung cancer harboring EGFR mutations: a review of the evidence. Onco Targets Ther..

[32] Camidge D.R., Pao W., Sequist L.V. (2014). Acquired resistance to TKIs in solid tumours: learning from lung cancer. Nat. Rev. Clin. Oncol..

[33] Normanno N., Maiello M.R., Chicchinelli N., Iannaccone A., Esposito C., De Cecio R. (2017). Targeting the EGFR T790M mutation in non-small-cell lung cancer. Expert Opin. Ther. Targets.

[34] Kanehisa M., Sato Y., Kawashima M., Furumichi M., Tanabe M. (2016). KEGG as a reference resource for gene and protein annotation. Nucleic Acids Res..

[35] Pao W., Miller V.A., Politi K.A., Riely G.J., Somwar R., Zakowski M.F. (2005). Acquired resistance of lung adenocarcinomas to gefitinib or erlotinib is associated with a second mutation in the EGFR kinase domain. PLoS Med..

[36] Chen X., Song X., Li J., Wang J., Yan Y., Yang F. (2024). Integrated proteomic, phosphoproteomic, and N-glycoproteomic analyses of small extracellular vesicles from C2C12 myoblasts identify specific PTM patterns in ligand-receptor interactions. Cell Commun. Signal..

[37] Very N., Lefebvre T., El Yazidi-Belkoura I. (2018). Drug resistance related to aberrant glycosylation in colorectal cancer. Oncotarget.

[38] Isaji T., Im S., Gu W., Wang Y., Hang Q., Lu J. (2014). An oncogenic protein Golgi phosphoprotein 3 up-regulates cell migration *via* sialylation. J. Biol. Chem..

[39] Zhao Y., Li Y., Ma H., Dong W., Zhou H., Song X. (2014). Modification of sialylation mediates the invasive properties and chemosensitivity of human hepatocellular carcinoma. Mol. Cell Proteomics.

[40] Seales E.C., Jurado G.A., Brunson B.A., Wakefield J.K., Frost A.R., Bellis S.L. (2005). Hypersialylation of β1 integrins, observed in colon adenocarcinoma, may contribute to cancer progression by up-regulating cell motility. Cancer Res..

[41] Liu J., Cao S., Imbach K.J., Gritsenko M.A., Lih T.-S.M., Kyle J.E. (2024). Multi-scale signaling and tumor evolution in high-grade gliomas. Cancer Cell.

[42] Lih T.M., Cho K.-C., Schnaubelt M., Hu Y., Zhang H. (2023). Integrated glycoproteomic characterization of clear cell renal cell carcinoma. Cell Rep..

[43] Perez-Riverol Y., Bai J., Bandla C., Garcia-Seisdedos D., Hewapathirana S., Kamatchinathan S. (2022). The PRIDE database resources in 2022: a hub for mass spectrometry-based proteomics evidences. Nucleic Acids Res..

