# Supplementary material for: Metal Ion-Enhanced ZIC-cHILIC StageTip for N-Glycoproteomic and Phosphoproteomic Profiling in EGFR-Mutated Lung Cancer Cells
Source: Mol Cell Proteomics. 2025 Mar 26;24(6):100957. doi: 10.1016/j.mcpro.2025.100957 (PMC12289526; doi:10.1016/j.mcpro.2025.100957)
Supplement: Supporting Information [file mmc9.pdf]

Supplementary Figure S1-S12

Metal ion-decorated ZIC-cHILIC StageTip Enables Simultaneous Profiling of N-Glycoproteome and Phosphoproteome in EGFR mutant Lung Cancer Cells

Yi-Ju Chen[1], Yan-Lin Chen[1,2], Kun-Hao Chang[1,3,4], Hsiang-Chun Cheng[2], Chiao-Chun Chang[2], Yu-Ju Chen[1,2,3*]

[1] Institute of Chemistry, Academia Sinica, Taipei, Taiwan
[2] Department of Chemistry, National Taiwan University, Taipei, Taiwan
[3] Molecular Science and Technology Program, Taiwan International Graduate Program, Academia Sinica, Taiwan
[4] Department of Chemistry, National Tsing-Hua University, Hsinchu, Taiwan

*** Corresponding author information**
Yu-Ju Chen
https://orcid.org/0000-0002-3178-6697

Institute of Chemistry, Academia Sinica, 128 Academia Road, Section 2, Nankang, Taipei 11529, Taiwan.
Email: yujuchen@gate.sinica.edu.tw

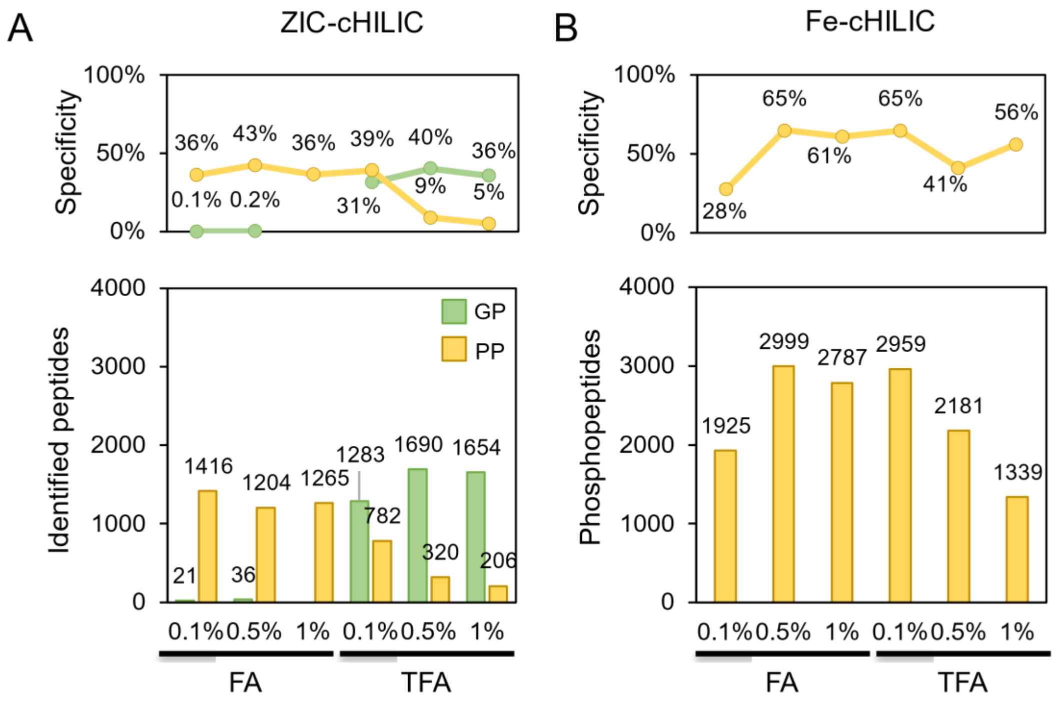

Supplementary Figure S1. Evaluation of different incubation buffers for ZIC-cHILIC and Fe-cHILIC StageTip. 100 µg of peptides from PC9 cell lysate were used to evaluate the performance in different incubation buffers. (A) The specificity and number of identified intact glycopeptides (GP) and phosphopeptides (PP) enriched by ZIC-cHILIC StageTip were shown in the line chart and bar chart respectively. (B) The specificity and identification results of phosphopeptide were further inspected in Fe-cHILIC StageTip with different incubation buffers.

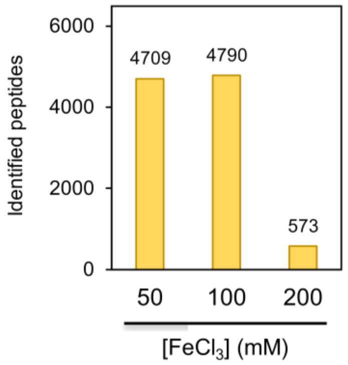

Supplementary Figure S2. Optimization of $FeCl_3$ concentration for Fe-cHILIC StageTip. The concentration of $FeCl_3$ with 50, 100 and 200 mM was used for reacted with ZIC-cHILIC. After washing by ddH_2O, 100 µg of peptides from PC9 cell lysate were loaded in the Fe-cHILIC StageTip to evaluate the performance of intact glycopeptides enrichment.

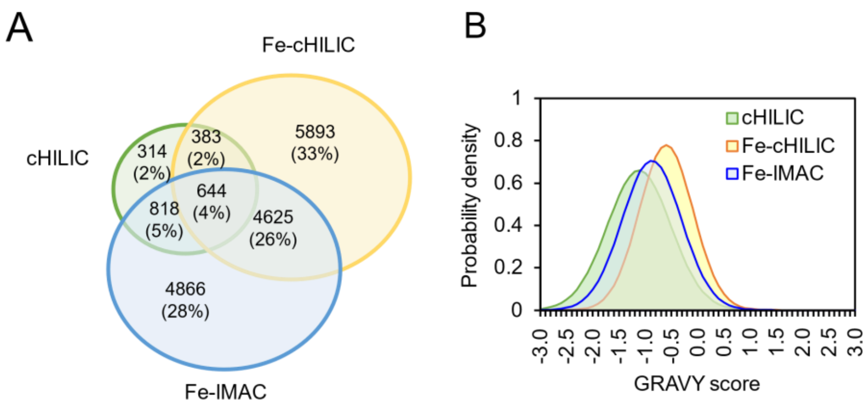

Supplementary Figure S3. Assessment of three enrichment methods for phosphopeptide. (A) Venn diagram representing the overlap of 17,543 phosphopeptides under ZIC-cHILIC, Fe-HILIC and Fe-IMAC. (B) Hydrophobicity of identified phosphopeptides enriched by ZIC-cHILIC, Fe-cHILIC, and Fe-IMAC were evaluated by gravy score.

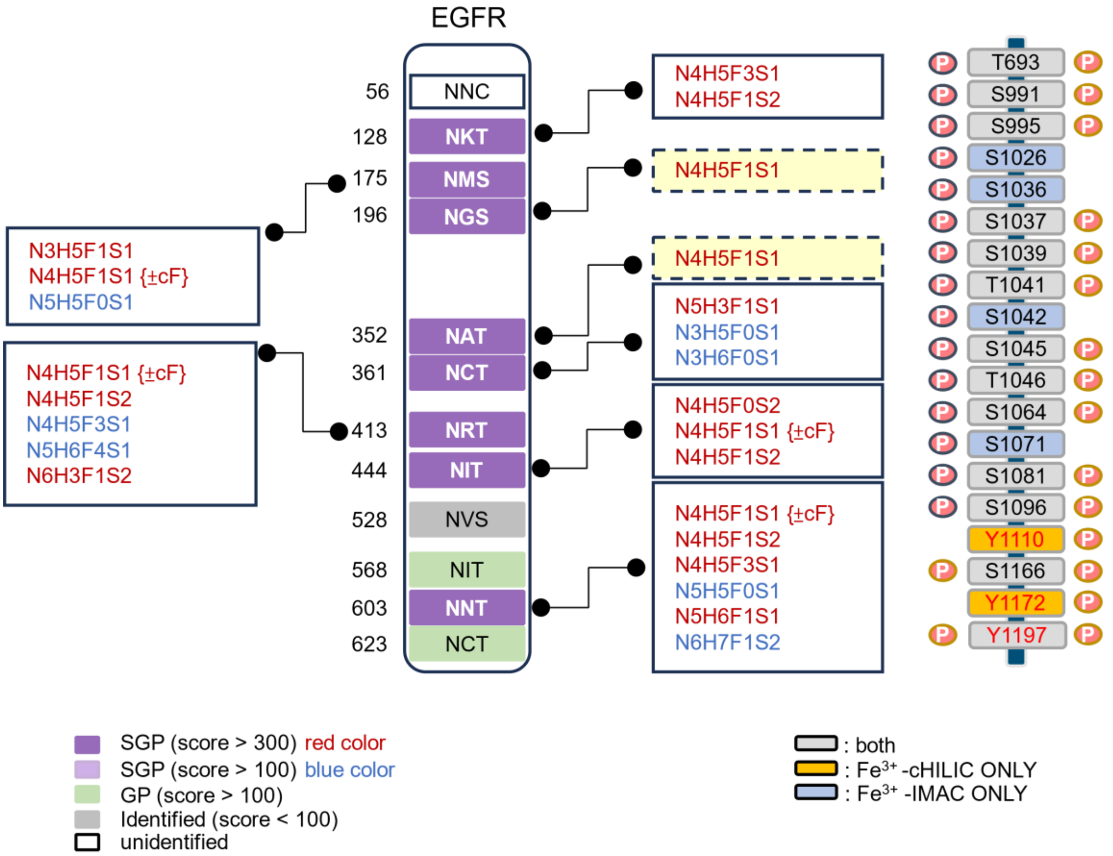

Supplementary Figure S4. N-glycoproteomic and phosphoproteomic profile on EGFR protein in PC9 cell. The identified N-glycosites, glycan composition, and phosphosites were annotated on EGFR protein. N, HexNAc; H, Hex; F,Fuc; S, NeuAc. cF, core-Fuc. P, phospho.

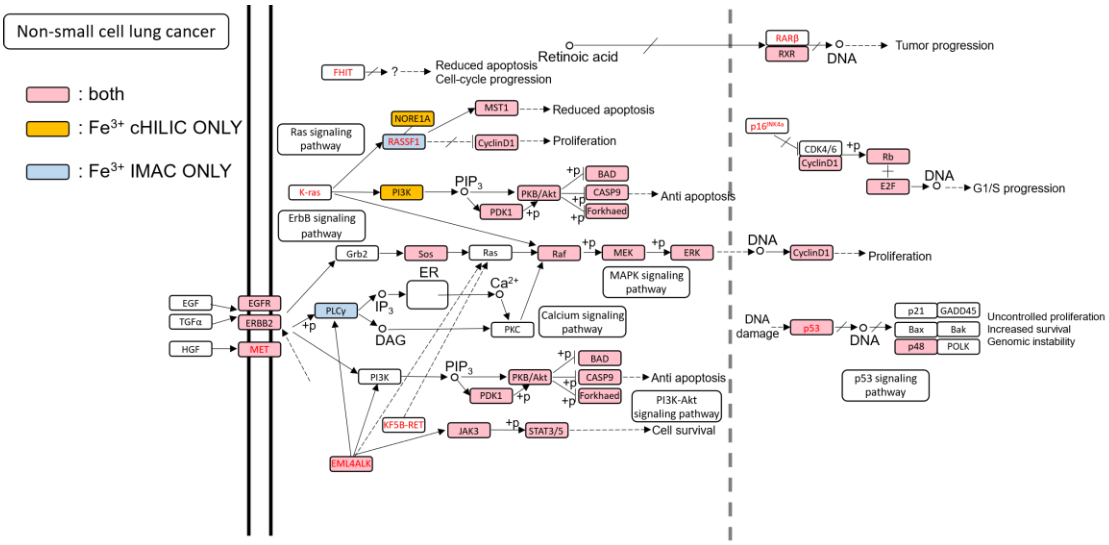

Supplementary Figure S5. Illustration of non-small-cell lung cancer (NSCLC) pathway by cHILIC, Fe-cHILIC, and Fe-IMAC StageTip strategy. The identified glycoproteins and phosphoproteins were marked in the NSCLC pathway.

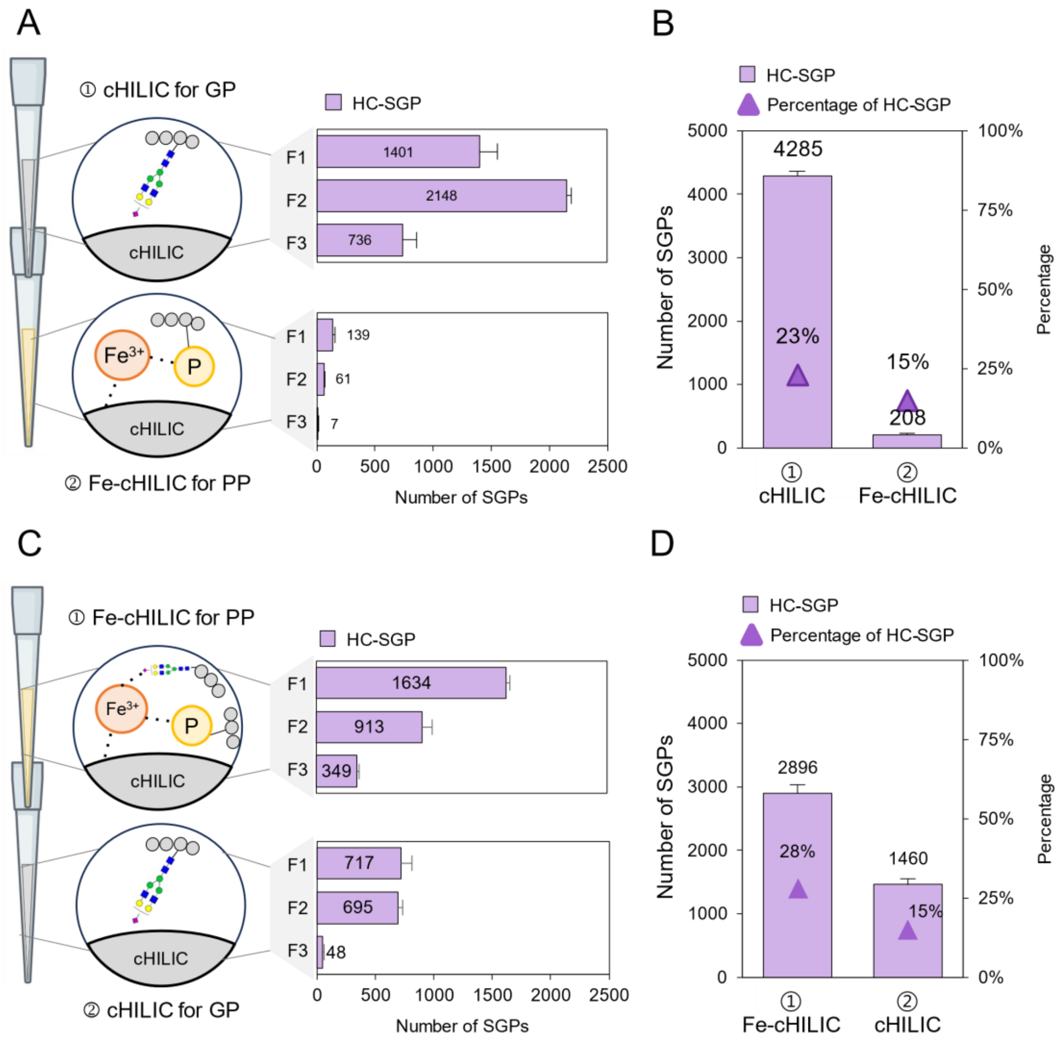

Supplementary Figure S6. Evaluation the performance of sialo-glycopeptides under different order of sequential experiment by cHILIC and Fe-cHILIC tandem tip. (A) Enrichment is performed in the order of ① cHILIC and ② Fe-cHILIC. Performance is shown on the number of identified sialo-glycopeptides (SGPs) and enrichment specificity of SGPs in the three fractions: F1 (70% ACN), F2 (65% ACN), F3 (60+55+50% ACN). (B) Summary of total number of SGPs and enrichment specificity (%) by combining three fractions. (C) Enrichment is performed in the order of ① Fe-cHILIC and ② cHILIC for SGPs. Performance is shown on the number and enrichment specificity of identified SGPs in the three fractions. (D) Summary of total number and enrichment specificity (%) of SGPs by combining three.

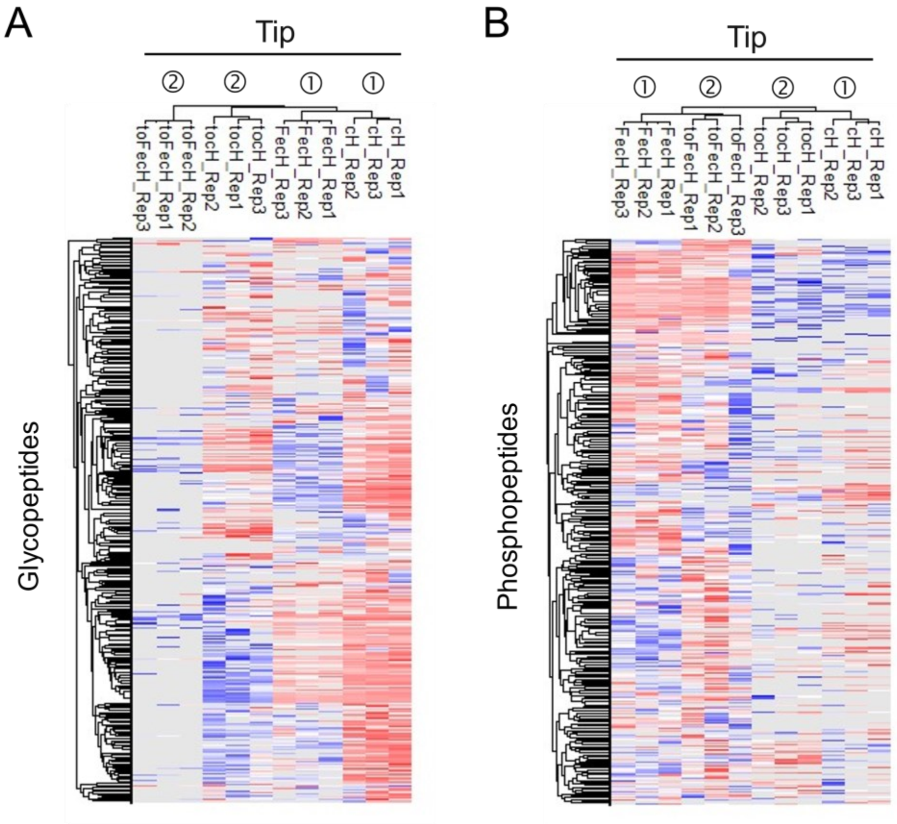

Supplementary Figure S7. Quantitative coverage of intact glycopeptides and phosphopeptides from different orders of tandem tip enrichment. (A) Overall abundance of (A) intact glycopeptides and (B) phosphopeptides between four different StageTips were presented by hierarchical clustering and heatmap. The heatmap shows the $\log_2$ scale of the expressed abundance of glycopeptides and phosphopeptides. The values were labeled with color from red to blue, representing intensity from high to low. Every row represents an individual peptide, and the column represents each sample. Tandem tip 1 - ♊top cHILIC StageTip (cH); ♋ bottom Fe-cHILIC StageTip (to FecH). Tandem tip 2 - ♊top Fe-cHILIC StageTip (FecH); ♋ bottom cHILIC StageTip (tocH). Rep, replicate number.

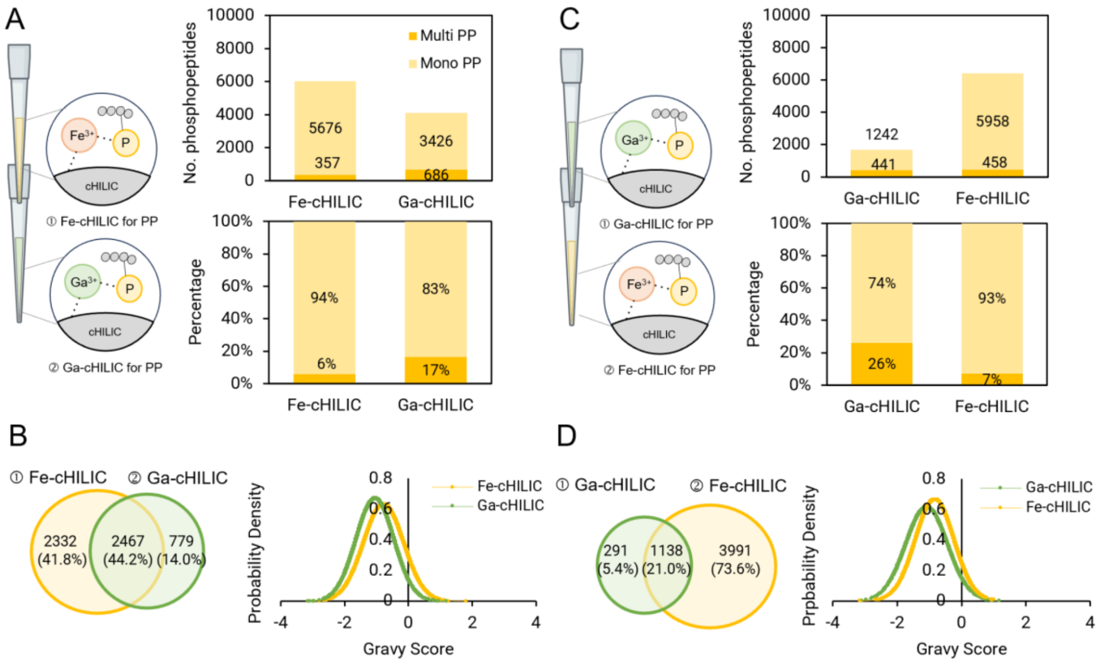

Supplementary Figure S8. Evaluation of phosphopeptide enrichment performance by Fe and Ga-cHILIC using 200 µg protein digest from PC9 cells. The comparison was performed by different orders of sequential enrichment using Fe and Ga. (A) Comparison of the number and percentage of multi- and mono-phosphopeptides (multi PP and mono PP) by first enrichment using Fe-cHILIC, followed by second enrichment using Ga-cHILIC. (B) Venn diagram showing the number and percentage of overlapping phosphopeptides enriched by Fe-cHILIC and Ga-cHILIC. Common PPs account for 44.2% (2,467 PPs). The GRAVY score shows the distribution of the hydrophobicity of the phosphopeptides. (C) Comparison of the number and percentage of multi- and mono- phosphopeptides (multi PP and mono PP) by first enrichment using Ga-cHILIC, followed by second enrichment using Fe-cHILIC. (D) Venn diagram showing the number and percentage of overlapping phosphopeptides in Ga-cHILIC and Fe-cHILIC. Common PPs account for 21.0% (1,138 PPs). The GRAVY score indicates the distribution of the hydrophobicity of the phosphopeptides.

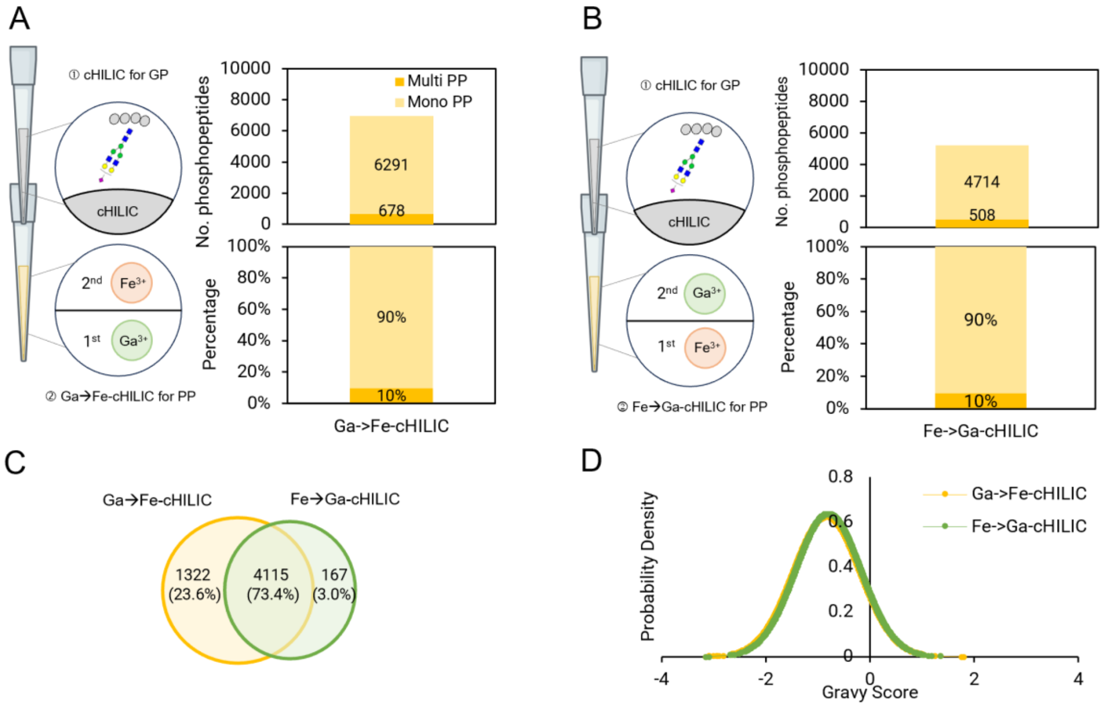

Supplementary Figure S9. Evaluation of dual-metals, doping both Fe and Ga in different packing order, in one tip. The performance was evaluated by using 200 µg protein digest from PC9 cells. (A) Comparison of the number and percentage of multi- and mono- phosphopeptides (multi PP and mono PP) by packing Ga-cHILIC first, then Fe-cHILIC (Ga->Fe-cHILIC). The identification number was based on the count of specific phosphosites. (B) Comparison of the number and percentage of multi- and mono- phosphopeptides (multi PP and mono PP) by packing Fe-cHILIC first, then Ga-cHILIC (Fe->Ga-cHILIC). (C) Venn diagram showing the number and percentage of overlapping phosphopeptides in Ga->Fe-cHILIC and Fe->Ga-cHILIC. Common PPs account for 73.4% (4,115 PPs). (D) The GRAVY value indicates the distribution of the hydrophobicity of the phosphopeptides enriched through Ga->Fe-cHILIC and Fe->Ga-cHILIC.

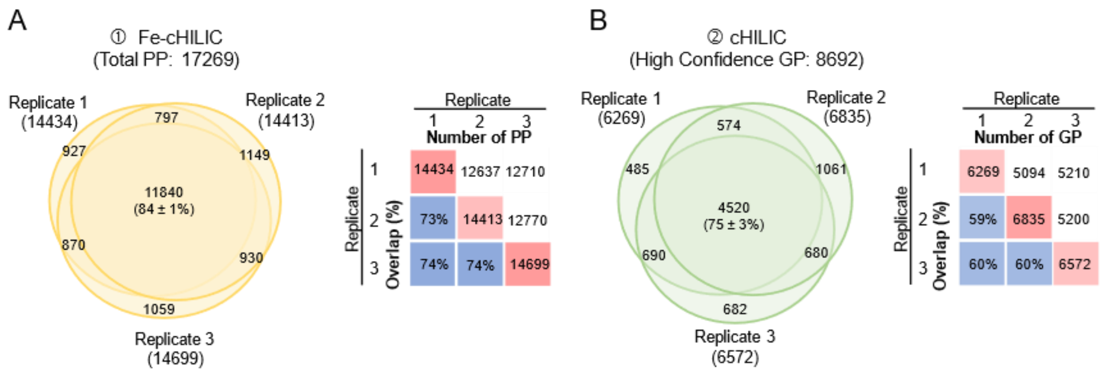

Supplementary Figure S10. Evaluation of reproducibility by triplicate sequential enrichment in the order of StageTip ♊ Fe-cHILIC for glycopeptides and ♋ cHILIC for phosphopeptides. (A) Venn diagram showed the number and percentage of overlapping phosphopeptides (PP) by Fe-cHILIC StageTip, which presented 84 ± 1% (11,840 common PPs) in triplicates. (B) Venn diagram showed the number and percentage of overlapping glycopeptides (GP) by ZIC-cHILIC StageTip, which presented 75 ± 3% (4, 520 common PPs) in triplicates.

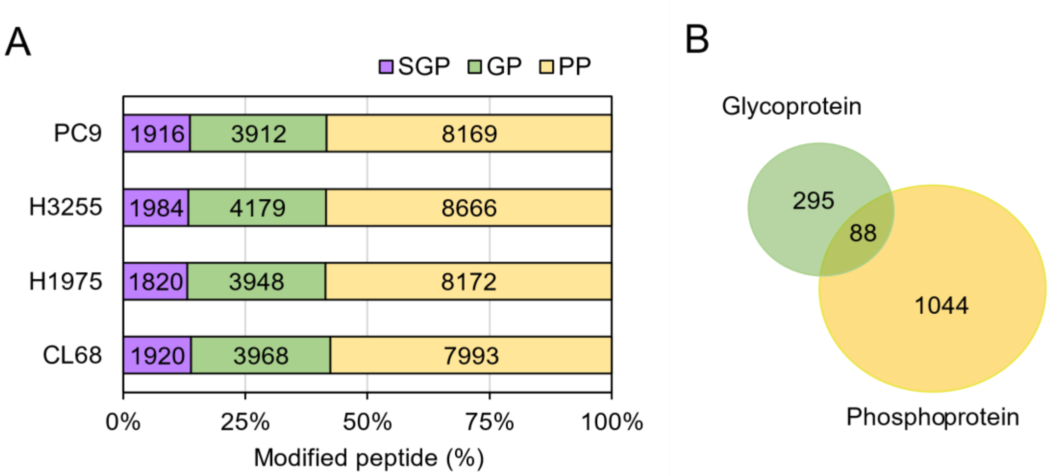

Supplementary Figure S11. Component distribution of intact glycopeptides and phosphopeptides in 4 different types of NSCLC cells. Equal amount of total peptides (200 µg) from 4 types of cell lysate was used for tandem tip enrichment. (A) The distribution of number and percentage of high confident intact glycopeptides, sialo-glycopeptides and phosphopeptides was similar in 4 samples. High confident GPs were selected by Byonic Score > 150, PEP2D < 0.01, and LogProb > 2. (B) Venn diagram present the proteins were identified and quantified with glycosylation, phosphorylation, or both.

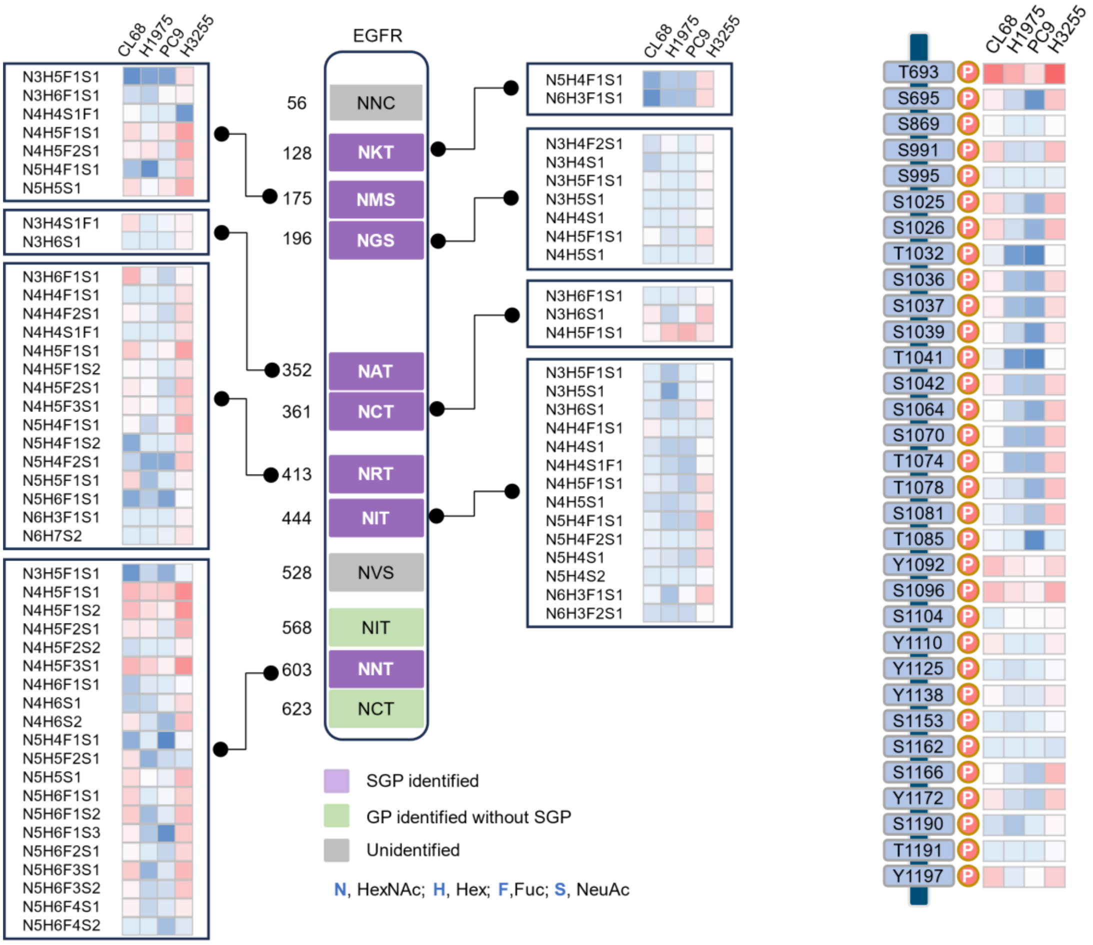

Supplementary Figure S12. N-glycoproteomic and phosphoproteomic profile on EGFR protein in 4 different EGFR subtypes of NSCLC cells. The differentially expressed N-glycosites, glycan compositions, and phosphosites were annotated on EGFR protein. N, HexNAc; H, Hex; F,Fuc; S, NeuAc. cF, core-Fuc. P, phospho.
